# Long-lasting effects of historical land use on the current distribution of mammals revealed by ecological and archaeological patterns

**DOI:** 10.1038/s41598-019-46809-1

**Published:** 2019-07-23

**Authors:** Keita Fukasawa, Takumi Akasaka

**Affiliations:** 1 Center for Environmental Biology and Ecosystem Studies, National Institute for Environmental Studies, 16-2 Onogawa, Tsukuba, Ibaraki 305-8506 Japan; 2 Conservation Ecology Lab., Obihiro University of Agriculture and Veterinary Medicine, Nishi 2-sen 11, Inadacho, Obihiro, Hokkaido 080-8555 Japan

**Keywords:** Biodiversity, Macroecology

## Abstract

Past land-use activity has massively altered the environment and vegetation over centuries, resulting in range contractions and expansions of species. When habitat recovery and species recolonization require a long time, the fingerprint of past land use can remain on the current distribution of species. To evaluate millennial-scale effects of land use in Japan, we explained the current ranges of 29 mammalian genera based on three types of archaeological land-use patterns (settlement, ironwork and kiln) considering potential confounding factors. The results indicate that archaeological human activity associated with ironwork and pottery production had severe negative effects on many genera of small and medium-sized mammals. Despite positive effects on some genera, the magnitudes were less than those of the negative effects. The relative importance of archaeological factors on small mammals was greater than those for medium- to-large mammals. The persistent imprint of past land-use patterns was non-negligible, explaining current mammalian diversity. Spatial ecological and archaeological information can provide meaningful insights into long-term socio-ecological processes, which are crucial for the development of sustainable societies in the Anthropocene.

## Introduction

Understanding the long-term consequences of land-use patterns on global biodiversity is one of the ultimate goals in ecological research and is crucial for the development of a sustainable society. Palaeoecological, anthropogenic and archaeological studies have provided evidence that ancient land-use activities massively altered macro-scale environments and vegetation over a period of thousands of years^1^. Examining current biodiversity patterns in relation to long-term human interventions thereby offers valuable insight into the temporal scale of land-use legacies^2^.

In particular, mammalian species have been affected by human activity for millennia. Although some invasive mammals expanded their range by adaptation to increasing transportation and land modifications by humans^3^, the range size and diversity of mammals have gradually decreased during the Holocene^4–6^, and historical anthropogenic pressure, such as over-exploitation and habitat destruction, were the major driving forces^6,7^. The rate of range contraction is related to body size^7^, which determines susceptibility to hunting pressure, growth, dispersal rates and habitat selectivity. If the range of a species contracts and subsequent recolonization is limited, the areas disturbed by past anthropogenic pressure exhibit “colonisation credit”^8^, characterised by fewer species than those in the historically undisturbed area. The slow recovery of habitat conditions (e.g. the recovery of old growth forest after disturbance) and persistent ecosystem alterations by regime shifts^9^ also mediate the long-term effects of past habitat destruction. Although the persistent imprint of the past environment is a non-negligible determinant of macro-ecological patterns^10–12^, the effect of millennial-scale land use on the current distributions of mammals is unclear.

Studying the relationship between past land use and current ecological patterns is a fundamental approach to evaluate legacy effects of past land use^2,13–15^, and archaeological sites provide useful information on ancient human activity^16^. Integrating spatial information in archaeology and biodiversity using a correlative approach provides meaningful insight into the effects of different land-use types and archaeological cultures on current biological patterns^17,18^.

Pre-modern Japan is considered one of the most successful cases of sustainable resource management, despite its very high population density^19^. Traditional land-use regimes created a cultural landscape called “satoyama”, harbouring diverse fauna^20^. However, other studies have shown that intensive land use involving tree cutting and agriculture have altered Japan’s ecosystem structure at a broad scale^21–24^. For example, energy-intensive industries, such as traditional ironwork (“*tatara”* in Japanese, Tate^25^) and pottery-making, caused the extirpation of fuelwood and subsequent alteration of ecosystem function^26^. Historically, the spatial patterns of human populations and land-use intensity were temporally heterogeneous owing to natural, cultural and political factors, such as the northward expansion of agriculture, development of highroads and capital relocation^27,28^. Thus, Japan provides a good case study for understanding the contributions of different archaeological cultures and lifestyles to current patterns. In Japan, over 400,000 records of archaeological sites are included in the Archaeological Sites Database (http://mokuren.nabunken.go.jp/Iseki/)^29,30^. This database records the latitude, longitude, historical period and type of archaeological sites, and spatiotemporal patterns in land-use intensity can be recovered.

In this study, we estimated long-lasting effects of archaeological land use on the current ranges of mammals in Japan and evaluated whether millennial-scale land-use intensities explain current ranges of mammals. To achieve these objectives, we examined the current distribution of 31 genera of mammals in Japan with respect to archaeological site patterns including three land-use types, settlement, ironwork and kiln, in six historical periods (Table 1). We also evaluated the relative contribution of archaeological factors and other environmental factors to the ranges of mammals and the relationship between these contributions and body size.

**Table 1:** Historical periods and archaeological site types considered in this study.

Period	Year^*a^	Archaeological site type		
		Settlement	Ironwork	Kiln
Jomon	ca. 12,000 BCE to ca. 300 BCE	○		
Yayoi	ca. 900 BCE to ca. 300	○		
Kofun	ca. 300 to ca. 700	○	○	○
Antiquity^*b^	592 to 1192	○	○	○
Feudal^*c^	1192 to 1573	○	○	○
Early modern^*d^	1573 to 1868	○	○	○

^*a^Jomon, Yayoi, Kofun and Antiquity are defined by the types of archaeological remains, such as stone tools and pottery, and architectural styles, and their boundaries overlap.

^*b^Consisting of the Asuka, Nara and Heian periods.

^*c^Consisting of the Kamakura, Nanbokucho, Muromachi and Sengoku periods.

^*d^Consisting of the Azuchi–Momoyama and Edo periods.

## Results

### Positive and negative effects of archaeological land use

**Figure 1:**
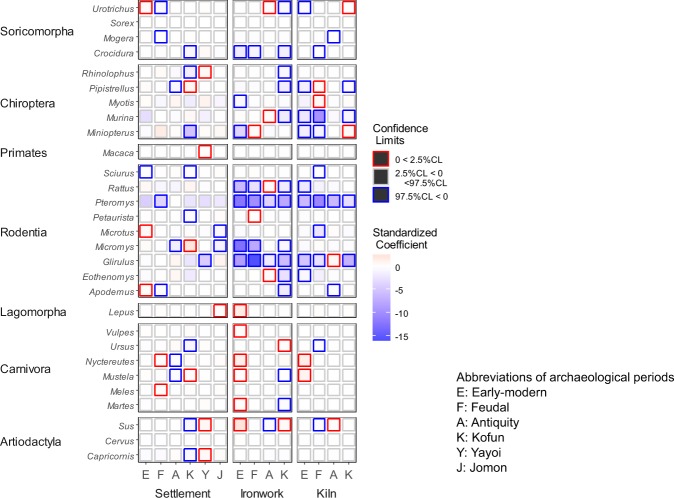
Tile chart of standardised coefficients of archaeological factors (posterior mean) and 95% credible limits for genera included in the analysis.

We estimated the effects of archaeological land use on 29 mammalian genera, except *Dymecodon* and *Chimarrogale* whose parameter estimates did not converge. We found that past land use had significant effects on multiple genera; however, the direction of the effect (i.e. positive or negative) differed among genera (Fig. 1). Estimated coefficients and the 95% CI for all genera are shown in Supplementary Table S1.

Ironwork had negative effects on a large proportion of mammals especially in the early-modern and Kofun periods; the 95% CIs for the coefficients were less than 0 for 7 and 13 genera, respectively. Strong negative effects of early-modern ironwork were detected for several genera of small to medium-sized mammals, such as *Crocidura*, *Rattus*, *Pteromys*, *Micromys* and *Glirulus*. These genera were also negatively affected by ironwork in multiple periods before the early-modern period, including the Kofun period, which is the earliest period with ironwork in Japan. We detected positive effects of early-modern ironwork on genera of medium-to-large mammals, such as *Lepus*, *Vulpes*, *Nyctereutes*, *Mustera*, *Martes* and *Sus* (Fig. 1). The magnitudes of positive coefficients were smaller than those of negative coefficients, on average.

We detected negative effects of kiln for a large proportion of genera, especially in early-modern and feudal periods; 95% CIs of coefficients were less than 0 for 9 and 11 genera, respectively (Fig. 1). We also detected effects of kiln in the Kofun period for several genera, such as *Pipistrellus*, *Murina*, *Pteromys* and *Glirulus*. Although there were positive effects of kilns for several genera with various body sizes, the magnitudes of the coefficients were smaller than those of negative coefficients, on average.

Although negative effects of settlement were detected for genera of various taxonomic groups and body sizes, the effect sizes were smaller, on average, than those of ironwork and kiln (Fig. 1). We detected positive effects in most periods, except antiquity. Effects of human activity in the oldest period, Jomon, were detected for several genera; negative effects were observed for two genera (*Microtus* and *Micromys*) and a positive effect was observed for *Lepus*.

### Difference in contributions of archaeological factors among body size classes

**Figure 2:**
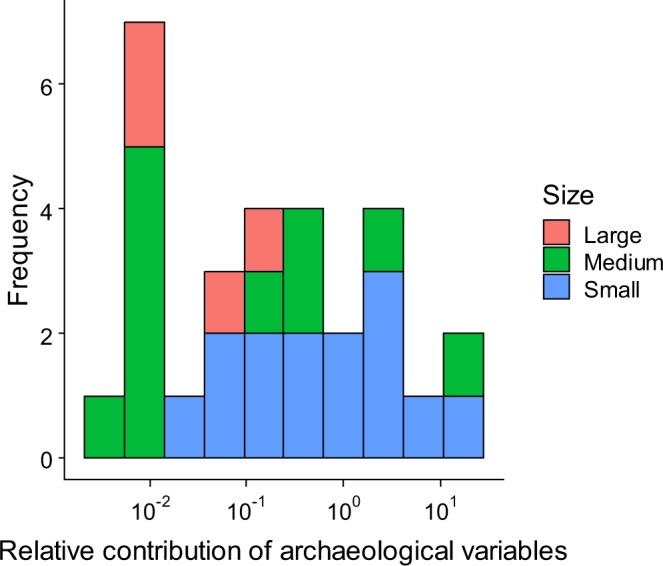
Summary of relative importance of archaeological factors^24^ in relation to body size.

The relative contributions of archaeological factors with respect to other explanatory variables, as determined by the proportion of the variance explained by linear predictor components^24^, varied among genera from 5.29 × 10^−3^ to 2.63 × 10^2^ (Fig. 2). Archaeological factors outperformed other factors for 8 out of 31 genera. Relative contributions were related to body size; the relative contribution of archaeological factors tended to be high for small mammals under microevolutionary models of Brownian motion and stabilising selection (phylogenetic linear mixed model, two-sided, *p* = 2.054 × 10^−3^ and 2.054 × 10^−3^, respectively).

## Discussion

Long-lasting effects of past land use on the current ranges of mammals could have several explanations: (1) long-term environmental changes caused by past land use^31,32^, (2) recruitment limitations after local extirpation^13^ and (3) regime shifts^33^. Although we were unable to identify the precise underlying process(es) using our approach, we observed a strong correlation between the macro-scale distribution of mammals and past human activities at an archaeological time scale, even when the effects of current land use were considered simultaneously. These results imply that human activity over the Holocene altered the geographical patterns of mammalian biodiversity, with long-lasting effects continuing until present.

Energy-intensive industries, such as ironwork and pottery-making, alter landscape structure and ecosystem functioning in Afro-Eurasia^34–36^. We found that such industries had strong effects on mammalian fauna. However, the effects were both positive and negative depending on the taxa. The negative effects of these industries were quite large and persisted for a long time. Such industries are dependent on large amounts of fuelwood, with the exploitation of forest resources causing the persistent degradation of forest ecosystems^37^. Moreover, frequent landform transformation occurred due to iron sand mining^38,39^. Despite detrimental negative effects, it should be noted that some genera responded positively to past energy-intensive industries. Intense human resource use results in the development of an open habitat consisting of semi-natural grassland and secondary forest, (i.e. ‘Satoyama’)^20^, which harbours a rich biota that is complementary to the intact habitat. However, this positive effect was weaker and less persistent than the negative effect (Fig. 1). These ecosystems are maintained by continuous human intervention, and a rapid change in landscape structure and loss of diversity are expected if these lands are abandoned^40^.

The substantial impact of archaeological factors on small mammals (Fig. 2) implies that traits associated with body size, such as limited dispersal^41,42^ and habitat specificity^43,44^, are crucial determinants of susceptibility to past land use over long time periods. Ground-dwelling small mammals have limited dispersal ability, and thus an “unpaid” colonisation credit^45^ would remain over centuries. A positive relationship between dispersal distance and body weight is common in terrestrial mammals^42^, and our results are consistent with the scaling rule. Small body size is also related to habitat specialisation due to energetic constraints^44^, and habitats for some small mammals are irreplaceable and not readily-recoverable. For example, small mammals, such as tree-roosting bats^46^ and the Japanese dwarf flying squirrel^47^, inhabit old-growth forests, which require a long time to recover after human disturbance. Although some small mammals became invasive and exhibited substantial range expansions, these species tended to have life history traits adapted to artificial open habitats and behavioural traits that allowed them to utilize human trade and transport to become established in human-dominated landscapes. Considering the scaling rules mentioned above, such traits observed in successful invaders would not be common in small mammals.

Although the relatively low importance of archaeological factors on medium-to-large mammals was not consistent with studies showing the rapid range contraction of large mammals in continental Eurasia^6,7^, our results are supported by zooarchaeological evidence. Tsujino *et al*.^48^ examined zooarchaeological and historical records for four medium-to-large mammals (*Cervus*, *Sus*, *Macaca* and *Ursus*) in Japan and showed that species ranges diminished substantially between the early-modern and modern periods. Over the past several decades, large mammals have undergone rapid range expansions in Japan^49^, and the recovery time after range contractions would be much shorter than the archaeological time scale. The difference between our results and those of previous studies on the continent can primarily be explained by the lack of large megafauna in Japan due to the mass extinction during the last glacial maximum (LGM) and subsequent geographical isolation. Megafauna such as *Alces alces*, *Bos primigenius*, *Mammuthus* and *Sinomegaceroides* were extinct in Japan during the LGM, and no immigration of megafauna in the Holocene is known^50^. Although Turvey *et al*.^7^ studied past range contractions of mammals in China and showed that a large body size is related to a high rate of range loss before 1900 CE, the largest and the most highly affected species, such as *Bubalus mephistopheles*, *Elephas maximus* and *Rhinoceros* spp., were not present in Japan during the LGM and Holocene^50^. Turvey *et al*.^7^ also showed that medium-to-large mammals in Japan, such as *Macaca*, *Nyctereutes* and *Ursus*, did not suffer severe range declines before 1900 CE. However, in *Cervus*, sika deer lost over 90% of their range in China, but red deer, which are larger than sika deer, maintained most of their range. When the largest mammals are excluded, the positive relationship between body size and susceptibility to past land use would not be clear, and the severe impact of past land use on the smallest mammals would be highlighted.

Although recent archaeological studies have shown a history of decline in mammalian diversity during the Holocene^6,7^, studies focused on small mammals (<1 kg) are limited. Zooarchaeological records of small mammals in the Holocene in Japan are not abundant^51,52^, and it is difficult to cross-check the effects of ironwork and pottery-making on small mammals at present. Although zooarchaeological studies can provide strong evidence for the coincidence of the ancient local extinction of animals and increases in anthropogenic pressure^53^, sampling can result in biassed estimates of the timing and rate of extinction, especially when the sample size is small^54^. In the future, large-scale studies of Holocene zooarchaeological data for small mammals are needed to confirm our hypotheses.

We used a grid-based dataset from a national mammal range survey generated by many specialists covering all of Japan and sampling bias is therefore expected to be small. However, missing grids would include both true absent grids and undetected grids. The inclusion of undetected grids can decrease statistical power for the logistic regression model^55^. In our study, the underestimation of regression coefficients could occur, especially for small-sized mammals, which are difficult to detect. This underestimation would lead to conservative results and therefore does not affect the validity of our findings; effects of archaeological factors were detected for small-sized species, even under such conservative settings. As is often the case in macroecological studies, another limitation of our study using grid-based distribution data is the inability to detect finer-scale phenomena. The most sensitive spatial scale differs among species and can affect the analysis of the relationships between body size and the relative importance of current and past land use. However, a global meta-analysis of the effects of the current landscape at multiple spatial scales has shown that the most important spatial scale is not proportional to body size or the scale of spatial use^56^, and the disproportionate underestimation of effects for small mammals is not likely. In Japan, Saito *et al*.^57^ showed that the optimal scale for the effect of urbanisation was a radius of 4–8 km or more for most mammals, regardless of body size, even if point-level data for mammals (camera traps) were used, and this scale was consistent with that in our study. Although the resolution of our data was better than some previous macroecological studies of mammals^58^, we admit that we cannot test the importance of current land use at a scale finer than 10 km × 10 km. This is a general limitation of data used for macroecological studies, emphasizing the need for local studies with finer resolution.

We used distributions of archaeological sites of different archaeological periods as proxies of past land use intensity owing to data limitation, but we should stress that caution is needed when interpreting archaeological periods. Generally, an archaeological period is associated with a lifestyle and culture, rather than an absolute timeframe. Despite extensive studies of the ranges of absolute ages of archaeological periods by radiocarbon dating^59^, cultures do not have distinct ages and often exhibit substantial overlap. Such characteristics of archaeological periods would make fine-scaled analyses of past anthropogenic effects difficult, and our results would reflect the effects of past land use by groups of people in the past sharing a lifestyle and culture. To improve precision, future research incorporating exhaustive databases of archaeological sites with radiocarbon dating is required. Another limitation of using archaeological sites as indices of past human activity is the potential for survey bias due to non-random survey designs. Social factors would affect the discovery rate and it is necessary to consider this source of bias in models of archaeological site distributions^60^. In Japan, the process of urban development might result in an increase in the rate of archaeological site discovery^61^. Although the spatial smoothing technique applied in this study would mitigate the effects of a local overabundance of archaeological sites in the crude data, a standard method to correct for survey bias is lacking. In this study, the effects of settlement were not strong for the mammalian genera, but the results might be influenced by survey bias. However, ironwork and kiln sites relevant to the principal findings of our study (i.e. effects of past energy-intensive industry on mammals and the relative importance of body size classes) would not be sensitive to such survey bias owing to the aggressive land modification and easy detection. Historically, steel and pottery industries formed major production areas that are well-known by their products^62,63^, and they tend to leave large well-preserved remains after repeated exposure to fire.

Understanding the history of ecological systems is important for land managers attempting to set appropriate conservation goals while considering the landscapes and for predicting the consequences of land management techniques^31,64^. However, little is known about the relationships between past human activities and ecosystems at an archaeological time scale. To gain a comprehensive understanding of the history of humans and ecosystems, it is therefore important to examine integrated information from various data sources, such as excavation records of archaeological remains, palaeoecological and zooarchaeological data, written historical records and the current status of species biodiversity. Our approach integrating spatial information for a range of mammalian species and archaeological data via statistical modelling provides a useful framework for examining feasible hypotheses related to long-term relationships between human history and biodiversity. The findings will be helpful in directing future studies of historical processes that have left a long-lasting footprint on the geographical patterns of current biodiversity.

## Methods

### Study area

**Figure 3:**
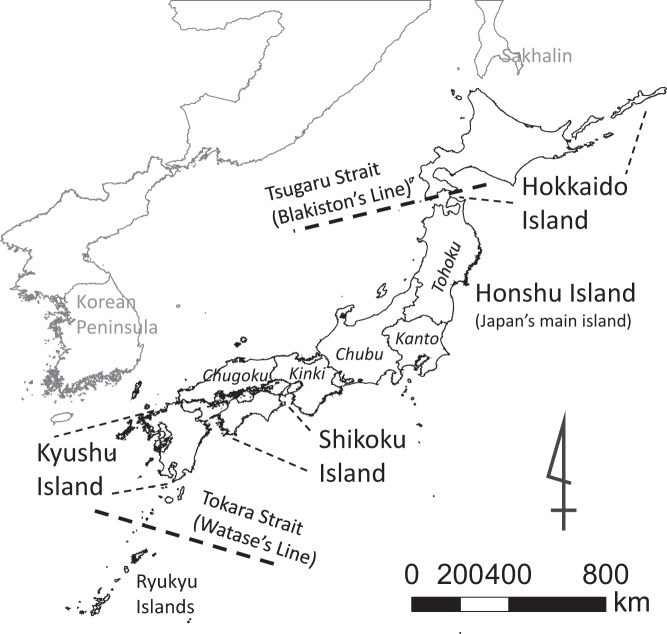
Map of the Japanese archipelago showing the Chugoku, Kanto, Kansai, Kyushu and Tohoku regions as well as the main islands and straits.

The Japanese Archipelago spans 20–45°N latitude. Japan is made up of four main islands, Hokkaido (83,457 km^2^), Honshu (227,970 km^2^), Shikoku (18,806 km^2^) and Kyushu (36,750 km^2^; Fig. 3), as well as over 6,000 additional islands, bringing its total land area to 378,000 km^2^. Although it extends from the subarctic to the subtropics, most of the archipelago lies in the temperate zone, with forests making up 67% of the landscape and thereby dominating the current vegetation.

Japan is a biodiversity hotspot^65^, with 104 terrestrial mammal species including 50 endemic (48.1% of the total) and 15 endangered species (CR and EN of IUCN Red List, 14.4% of the total). The islands of Honshu, Shikoku and Kyushu can be considered a single geographical unit in terms of mammalian biogeography; they are subdivided by shallow marine straits that formed no more than 8,000 years ago^66^. Two major biogeographical boundaries dissect Japan: the Tsugaru Strait between Hokkaido and Honshu, known as Blakiston’s Line, and the Tokara Strait between Kyushu and the Ryukyu Islands, known as Watase’s Line (Fig. 3)^66,67^. These boundaries define the range limits for many species of land mammals in Japan^66^.

Table 1 gives a summary of the Japanese historical periods considered in this study. In the Jomon period, settlements were present^68^. Although the Jomon people mainly depended on hunting-fishing-gathering for food, domesticated plants, such as millet and bean, were also utilized^69^. In this period, wildfires were frequently induced by humans^23^. Full-scale agriculture centred on paddy field farming began in the Yayoi period^70^ and is the major agricultural form to date. From Yayoi to antiquity, immigration from the continent and the spread of agriculture increased the population of western Japan^27,71^. For the feudal period, there is no reliable estimate of population size, but it is thought that there was gradual growth until the beginning of the early-modern period owing to improvements in agricultural techniques and trading^27^. In the early-modern period, although the development of new fields accelerated population growth, stagnation occurred during four serious famines (1640s, 1730s, 1780s and 1830s)^27,72^. In this period, settlements along roads developed as a result of the improved mobilisation of materials and humans.

Ironwork technologies were introduced from continental Asia in the Kofun period, and infrastructures of these traditional industries were later found in archaeological remains. Few bronze tools were found before the Kofun period, and it is thought that Japan lacked a bronze age. Although traditional ironworking reached its height in the early-modern period, it ceased during industrialization and was replaced by modern industries dependent on fossil fuels^73^.

Pottery-making technology using a kiln was introduced in Japan from the continent during the Kofun period^63^. In the feudal period, several pottery production areas in mid-west Honshu expanded production, with distribution extending far outside the area^74^. In the beginning of the early-modern period, Korean potters left after the Japanese invasion of Korea (1592–98), when porcelain production technology was introduced to Japan and kilns were established in Kyushu. Products were exported to Southeast Asia and Europe^75^. Although woody fuel was used for traditional pottery and porcelain production until the early-modern period, it was mostly replaced by fossil fuels during modernization^76^.

### Distribution data for mammals

A national survey of the current ranges of mammals in Japan was conducted by the Ministry of the Environment as a part of the Fifth National Survey on the Natural Environment (1993–1998) in cooperation with 3,433 volunteers nationwide. This survey is the most recent national distribution survey covering all mammalian species in Japan. In the survey, researchers, including specialists working at wildlife preserves as well as field scientists, reported occurrence records for each species. Researchers were nominated by a science committee to cover the entire country. Past distribution records and specimen records were also collected. These records were aggregated into the Second Standard Grid (SSG) of Japan, a national grid system with an approximately 10 km^2^ grid pattern (7.5° in longitude, 5° in latitude). The distribution maps were reviewed by independent specialists in each taxon and questionable records were deleted. Distribution maps were presented at SSG resolution with the most recent recorded date for each grid^77^. In our study, recent distribution records whose most recent date was after 1990 were used for statistical analysis. The nomenclature followed Ohdachi *et al*.^78^.

Aside from the effects of human activities, biogeographic processes, such as geographical isolation, speciation, interaction among species and biotic responses to the environment, are also crucial determinants of geographical distributions of organisms. To fully infer the effects of land use on mammalian distributions, the effects of these confounding factors on parameters related to mammalian distributions need to be eliminated. In Japan, many congeneric species show mutually exclusive distribution patterns indicating allopatric speciation or competitive exclusion (e.g. *Mogera* spp.^79^). In this study, distribution data for congeneric species were pooled to omit the effects of these interactions and to limit the scope of the study to genus-level phenomena. Ryukyu Islands were omitted from our analysis owing to their distinct biogeographical background from that of mainland Japan. For the same reason, Hokkaido was excluded from the analysis of genera with no occurrence records in Hokkaido. Genera endemic to Hokkaido were also omitted because the spatial variation in historical land use intensity and the physical environment of Hokkaido alone were too small for analyses. A total of 38 genera of native land mammals satisfied these conditions, seven of which—*Euroscaptor* (Soricomorpha), *Eptesicus*, *Nyctalus*, *Vespertilio*, *Barbastella*, *Plecotus* and *Tadarida* (Chiroptera)—had fewer than 30 presence records and were subsequently eliminated from further analyses. The 31 genera analysed included six genera of Soricomorpha, five of Chiroptera, one of Primates, six of Carnivora, three of Artiodactyla, nine of Rodentia and one genus of Lagomorpha (Supplementary Table S1). Distribution maps are shown in Supplementary Fig. S1. The adult size and food habit of each genera were obtained from Ohdachi *et al*.^78^ and taxa were assigned to three size classes (small, medium and large) according to Prothero^80^, where “small” is less than 100 g, “medium” is between 100 g and 10 kg and “large” is over 10 kg. The size of genera is shown in Supplementary Table S1.

### Archaeological land-use factors

Six historical periods discernible from features of archaeological sites were considered: (1) the Jomon (ca. 12,000 BCE to ca. 300 BCE), (2) Yayoi (ca. 900 BCE to ca. 300 CE), (3) Kofun (ca. 300 to ca. 700), (4) antiquity (592–1192), (5) feudal (1192–1573) and (6) early modern (1573–1868) periods. The density of archaeological sites was used as an index of ancient land-use intensity prior to the early-modern period. The Archaeological Sites Database (http://mokuren.nabunken.go.jp/Iseki/ (in Japanese)^29,30^, maintained by the Nara National Research Institute of Cultural Properties, Japan, has over 400,000 records of archaeological sites found in Japan. In Japan, every prefectural and municipal government has a section that collects information on archaeological sites for excavation in accordance with the Cultural Assets Preservation Act (1949), and many excavation surveys have been conducted throughout the country. This database is an exhaustive collection of excavation survey reports in Japan and includes information about archaeological sites, including the latitude, longitude, historical period and type of site. Three types of land use that can be distinguished by the characteristics of archaeological remains were considered: (1) settlements, (2) ironwork and (3) kilns for pottery-making.

The numbers of archaeological sites contain measurement noise due to stochasticity in the process of site discovery. When using such data as an index of ancient land use, it is necessary to filter the measurement noise and estimate the spatial gradient of land-use intensity. Accordingly, the number of archaeological sites by era and type was counted within each grid cell to match the spatial resolution to the mammalian distribution data. An intrinsic conditional autoregressive (CAR) model^81^ was then used for the spatial smoothing of archaeological sites. The Supplementary Methods include technical details related to this procedure. The estimated mean number of archaeological sites was used as the explanatory variable in the following analysis. Supplementary Figs S2, S3 and S4 provide maps of the indices of historical land use used in this study.

### Physical environment and current land-use factors

Six physical environmental factors and two current land-use factors were also included as explanatory variables: mean annual temperature, annual precipitation, precipitation in summer (July to September), snow depth, elevation, topographic roughness, urban area and agricultural land area. The four climatic factors—mean annual temperature, annual precipitation, precipitation in summer and snow depth—were obtained from Mesh Climatic Data 2000^82^. The two topographic factors, namely, elevation and topographic roughness, were defined by the average and standard deviation of a 1-km digital elevation model aggregated into SSG and were calculated using ArcGIS 10.0 (ESRI, Inc., Riverside, CA, USA).

The current land-use factors were obtained from Land Use Fragmented Mesh Data (http://nlftp.mlit.go.jp/ksj-e/jpgis/datalist/KsjTmplt-L03-b.html) in 1987, which was developed by the Ministry of Land, Infrastructure, Transport and Tourism, Japan. Areas of each land-use type were calculated for all SSG cells using ArcGIS.

### Past geoclimatic events

Past geoclimatic events can affect the ranges of mammals^12,83^ and should be considered confounding factors when we estimate the effects of archaeological land use. In the Holocene, Japan experienced two major geoclimatic events, the Younger Dryas Stadial^84^ and Mid-Holocene Climate Optimum^85^, with potential effects on the ranges of mammals. Variables associated with these events were included as confounding factors. The Younger Dryas Stadial at about 12,860–11,640 yr BP was characterised by a sudden drop in temperature^84^ and a dry climate^86^ that resulted in a change in vegetation in Japan^87^. A warm and wet climate prevailed in Japan during the Mid-Holocene Climate Optimum around 5,500–6,000 yr BP. In addition to warming, a global sea level rise of 2–10 m (Mid-Holocene transgression) occurred and coastal landforms were remarkably altered in Japan^88^. Our analysis included 2.5-minute downscaled mean annual temperature and annual precipitation in the Younger Dryas Stadial and Mid-Holocene^89^, reconstructed based on daily simulation output from the Community Climate System Model ver. 3^90^ and aggregated into SSG by taking the average of 2.5-minute grid values. Correlations among the current, Mid-Holocene and Younger Dryas climatic variables were high; Pearson’s correlation coefficients for mean annual temperature and annual precipitation were 0.985–0.999 and 0.838–0.997, respectively. To avoid failures in parameter estimation, we used the difference from the current value for Mid-Holocene climatic variables. For the same reason, the difference from the Mid-Holocene was used for the Younger Dryas. This process affects neither the parameter estimates for archaeological factors nor their relative contributions to mammal range patterns. As a confounding factor in the Mid-Holocene transgression, a binary variable indicating whether each SSG contains the submerged area was included^91^.

### Statistical analysis

Land-use patterns during different historical periods can be correlated because the process of land-use change depends on past patterns^92^, and historical periods that potentially influence the distributions of taxa should be considered in statistical analyses to tease out the effects of different historical periods. Explanatory variables included the archaeological land-use indices for settlements in six historical periods, ironwork and kilns of four historical periods, the six physical environmental factors, the two present land-use types and five past geoclimatic factors. All explanatory variables were included in multiple regression models to tease out partial contributions of archaeological land use types in different archaeological periods. Pearson’s correlation coefficients for relationships among explanatory variables ranged from −0.692 to 0.879.

For the statistical analysis of species distribution data, spatial autocorrelation should be considered to avoid type I errors for regression coefficients^93^, and a logistic regression model with spatial random effect implemented by intrinsic CAR model was used for grid-based data^81,94^. This model can accommodate spatially correlated random effects representative of unquantifiable factors and often yields accurate parameter estimates of focal factors^95^. In an intrinsic CAR model, spatial correlation of random effects is represented by the prior distribution for each grid cell whose mean is equal to average of the adjacent cells (i.e. the prior distribution was conditional on adjacent cells). It acts as a penalty to constrain neighbouring random effects to take similar values, with a smooth surface of spatial random effects to trace spatial trends of observations. This approach has three practical advantages: the assumption of the independence of samples is not required, type I errors due to autocorrelation are prevented, and spatial random effects improve the model fit by representing residuals that are not explained by fixed effects.

An intrinsic CAR model with Bernoulli observation error and logit link was fitted to the presence/absence data for each genus using Eq. (1):

1 $${\rm{Logit}}\,({\rm{P}}({y}_{i}=1))=\alpha +{{\bf{X}}}_{i}{\boldsymbol{\beta }}+{\rho }_{i}$$

where *y*_i_ is the presence/absence of a genus in the *i*^th^ cell, α is the intercept, **β** is the vector of the regression coefficients, **X**_*i*_ represents the explanatory variables and *ρ*_*i*_ is a spatially structured random effect. Prior to model fit, all the explanatory variables were standardised (i.e. scaled to mean = 0 and variance = 1) to allow the interpretation of the regression coefficients as an increase in prevalence (in logit scale) per 1 SD increase in the explanatory variable. The prior of *ρ*_*i*_ is represented by the conditional distribution of all elements of **ρ** except *ρ*_*i*_ (denoted *ρ*_-*i*_) in Eq. (2):

2 $${\rho }_{i}|{\rho }_{-i} \sim N(\frac{\sum _{j\in {\delta }_{i}}{\rho }_{j}}{{n}_{i}},\frac{{\sigma }_{\rho }^{2}}{{n}_{i}})$$

where *σ*_*ρ*_^2^ is the conditional variance of *ρ*_*i*_, *δ*_*i*_ is the set of labels for neighbours in area *i* and *n*_*i*_ is the length of *δ*_*i*_. The approximate posterior distribution was estimated by integrated nested Laplace approximation implemented in INLA (http://www.r-inla.org/)^96^. An inverse-gamma distribution with shape parameter 0.5 and inverse scale parameter 0.0005 was applied, as suggested by Kelsall and Wakefield^97^, as the prior distribution of *σ*_*ρ*_^2^.

To evaluate the contributions of archaeological factors to mammalian ranges relative to other factors, relative dispersion of components of the fit (RDCF)^24^ was applied, which is the ratio of the variances of the contributions of two groups of explanatory variables to the log-odds defined as follows:

$$\omega =\frac{{({{\bf{X}}}_{1}{{\boldsymbol{\beta }}}_{1})}^{T}{{\bf{X}}}_{1}{{\boldsymbol{\beta }}}_{1}}{{({{\bf{X}}}_{2}{{\boldsymbol{\beta }}}_{2})}^{T}{{\bf{X}}}_{2}{{\boldsymbol{\beta }}}_{2}}$$

where **X**_**1**_ and **X**_**2**_ are matrices of explanatory variable group compared and **β**_**1**_ and **β**_**2**_ are the corresponding vectors of regression coefficients. In this study, RDCF of archaeological factors against the other factors was calculated. *ω = *1 indicates that half of the observed variance is explained by archaeological factors. To evaluate the relationship between RDCF and body size, a phylogenetic linear mixed model considering inter- and intra- taxon variation^98^ was used. For our study, it is described by the following form:

$${\boldsymbol{\omega }} \sim {\rm{MN}}({\alpha }_{0}+{\alpha }_{1}{\bf{z}},{\boldsymbol{\Sigma }}),$$

where **ω** is a vector of ln(RDCF) of the genera estimated, *α*_0_ is the intercept, *α*_1_ is the regression coefficient of body size class and **z** is a vector of binary variables indicating whether the genera are classified as “small”. **Σ** is the inter- and intra- taxon covariance structure (the latter also includes measurement error) and is the sum of the inter-taxon variance-covariance matrix **Σ**_**S**_ and diagonal matrix of intra-taxon variance **Σ**_**M**_ = *v*_*M*_**I**. We considered two covariance structures for **Σ**_**S**_ corresponding to the microevolutionary models of Brownian motion and stabilising selection. Under Brownian motion, elements of the variance-covariance matrix, Σ_*Sij*_, equal *γC*_*ij*_ where *γ* (>0) is a parameter determining the strength of phylogenetic dependence and *C*_*ij*_ is the shared branch length (i.e. the length between the root and common ancestor) for taxa *i* and *j*. The stabilising selection model assuming that taxa with extreme phenotypic values are more likely to evolve toward less extreme values results in a variance-covariance structure Σ_*Sij*_ = *γ*exp(−*kD*_*ij*_), where *γ* and *k* are parameters and *D*_*ij*_ is the phylogenetic distance (i.e. the internode length to the common ancestor) between taxa *i* and *j*^98^. The divergence time estimates included in the mammalian supertree were obtained from Binida-Emonds *et al*.^99^.

## Supplementary information

Supplementary Information for “Long-lasting effects of historical land use on the current distribution of mammals revealed by ecological and archaeological patterns”

## Data Availability

The dataset analysed in the present study is available in Open Science Framework: https://osf.io/mjb2g/.
