# Supplementary material for: Long-lasting effects of historical land use on the current distribution of mammals revealed by ecological and archaeological patterns
Source: Sci Rep. 2019 Jul 23;9:10697. doi: 10.1038/s41598-019-46809-1 (PMC6650404; doi:10.1038/s41598-019-46809-1)
Supplement: Supplementary file 1 — Supplementary Information for “Long-lasting effects of historical land use on the current distribution of mammals revealed by ecological and archaeological patterns” [file 41598_2019_46809_MOESM1_ESM.pdf]

Supplementary Information for “Long-lasting effects of historical land use on the current distribution of mammals revealed by ecological and archaeological patterns”

Keita Fukasawa and Takumi Akasaka

Contents

Supplementary Methods Technical details for estimating indices of ancient land use using intrinsic CAR models.

Supplementary Figure S1 Distribution maps of mammalian genera analysed.

Supplementary Figure S2 Maps of settlement during six historical periods.

Supplementary Figure S3 Maps of ironwork during four historical periods.

Supplementary Figure S4 Maps of kiln during four historical periods.

Supplementary Table S1 Traits and posterior mean (posterior standard deviation, lower and upper 95% credible limit) of the regression coefficients for each genus.

Supplementary Methods Technical details for estimating indices of ancient land use using intrinsic CAR models.

To obtain indices of ancient land use, we applied an intrinsic CAR model with the loglinear Poisson model. In the intrinsic CAR model, the spatial correlation of random effects is represented by the prior distribution for each grid cell whose mean is equal to average of the adjacent cells, (i.e. the prior distribution was conditional on adjacent cells). It acts as a penalty to constrain neighbouring random effects to take similar values, with a smooth surface of spatial random effects to trace spatial trends in observations. When it is applied to the smoothing of land use indices, it acts as a filter to tease out meaningful trends from observation noise. The number of archaeological sites in the *i*th grid Y_i is count data assumed to have a Poisson distribution with a mean λ_i, which was used as an index of ancient land use. To estimate spatially smooth λ_i, we assumed that λ_i is represented by the spatial random effect model shown in Eq. (A1):

$$\log (Y_i) = \alpha + \rho_i \qquad \text{(A1)}$$

where α is the logarithmic mean and ρ_i is the spatial random effect with an intrinsic CAR prior identical to Eq. (2) in the "Statistical analysis" section of the main text. We applied an inverse-gamma distribution with shape parameter 0.5 and inverse scale parameter 0.0005 as the prior distribution of the conditional variance of the intrinsic CAR prior. Similar to the ancient land use indices, the posterior median was calculated using WinBUGS 1.43 (Lunn *et al.*, 2000).

Supplementary Figure S1 Distribution maps of mammalian genera analyzed.

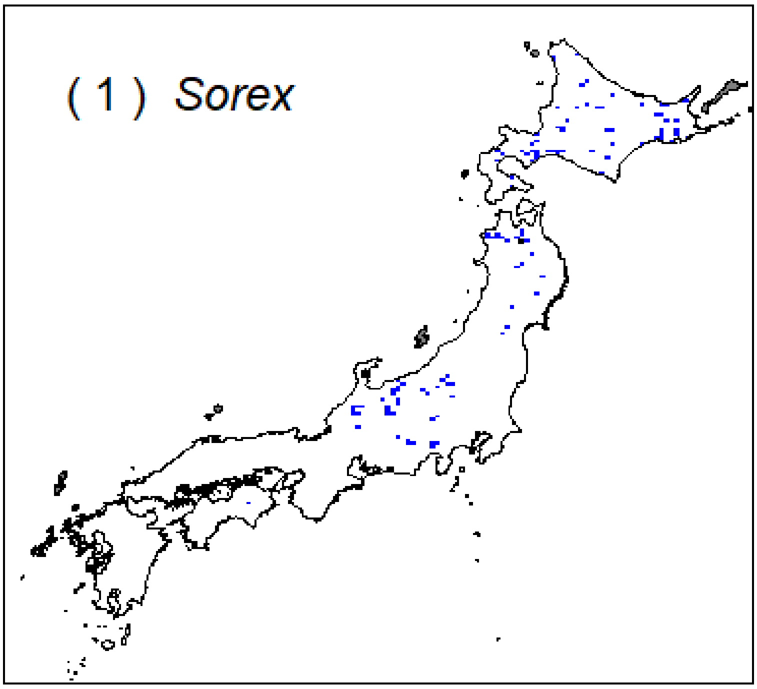

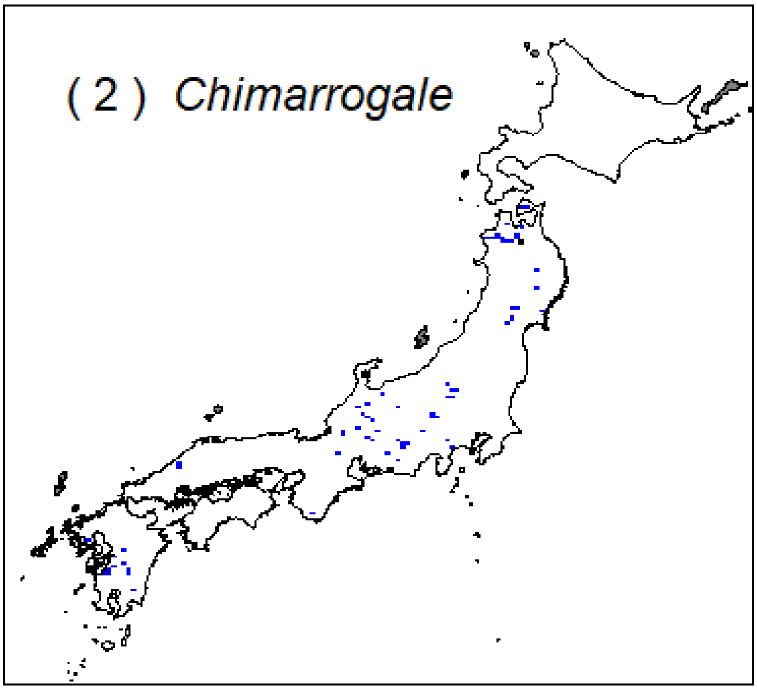

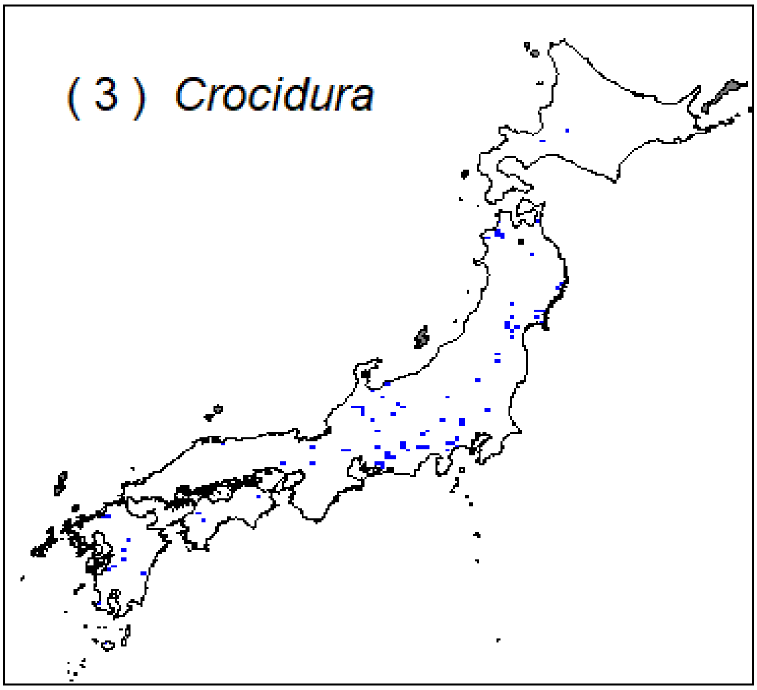
(3) *Crocidura*

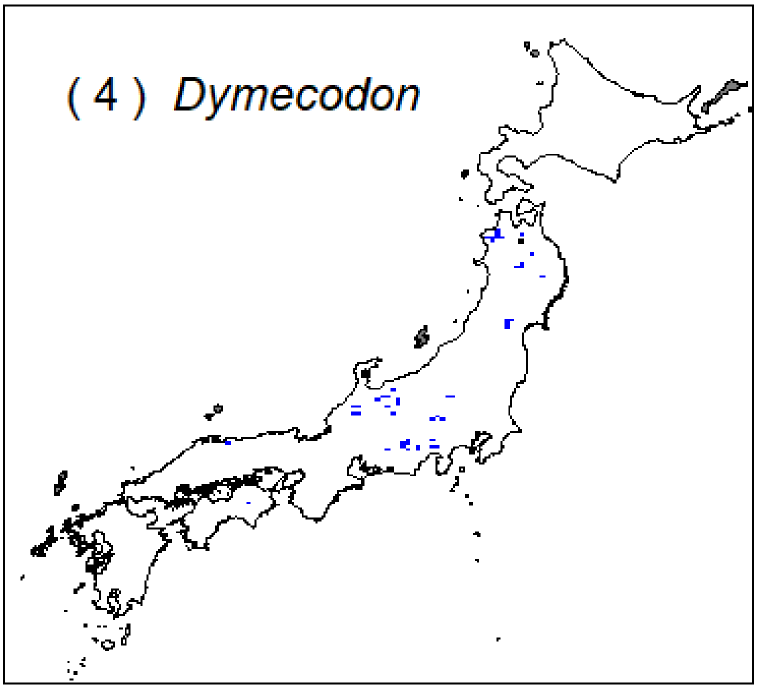
(4) *Dymecodon*

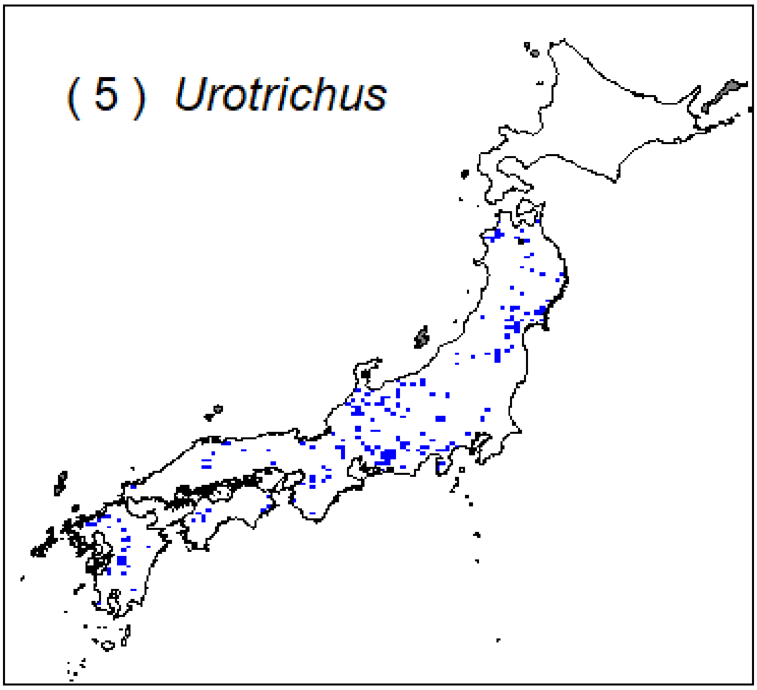
(5) Urotrichus

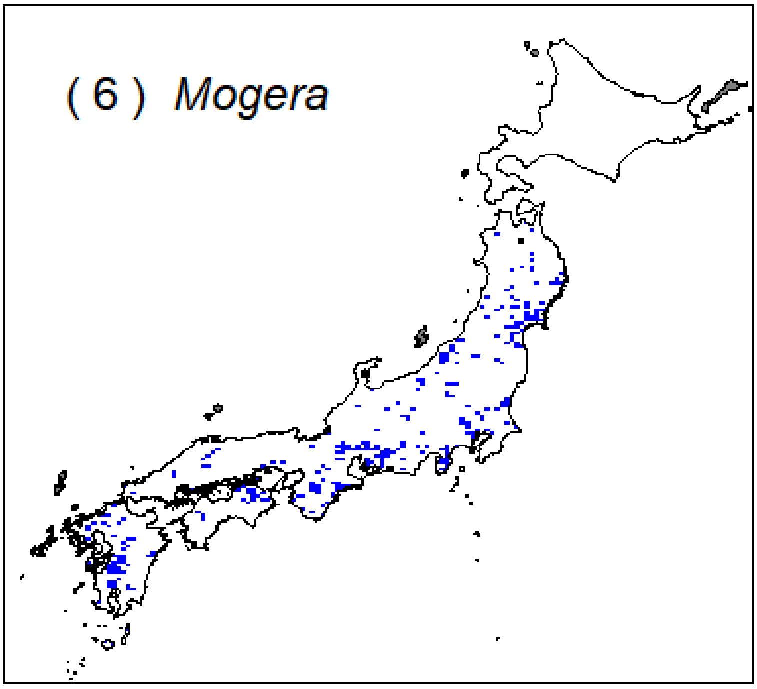
(6) Mogera

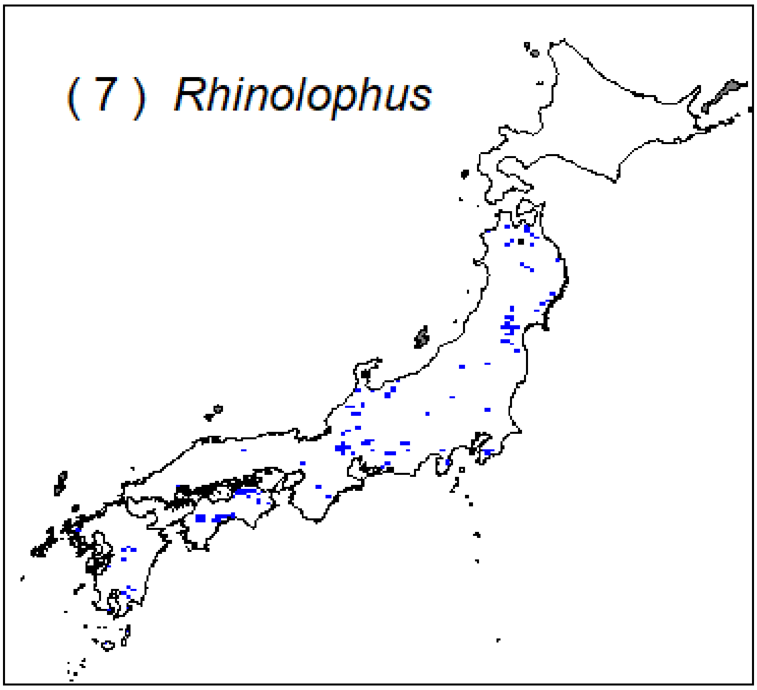
(7) *Rhinolophus*

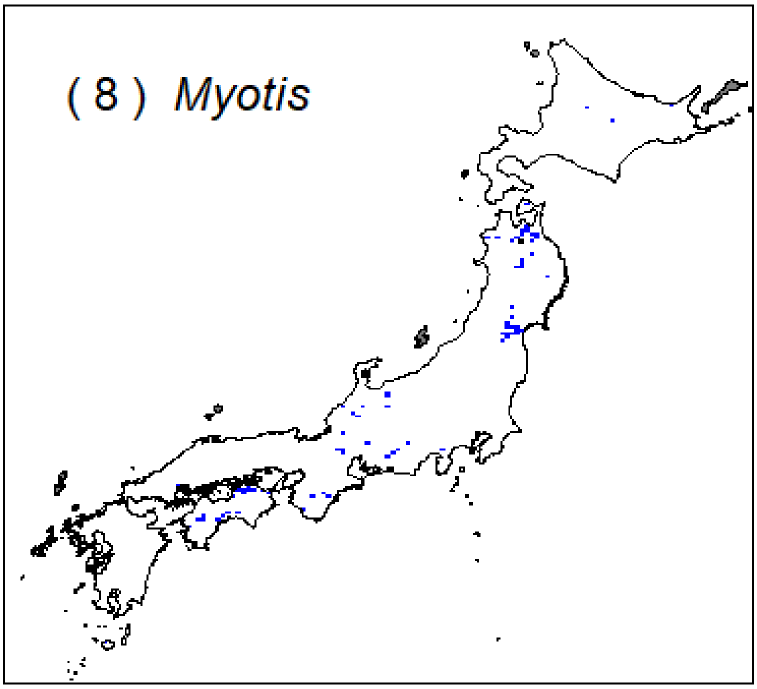
(8) *Myotis*

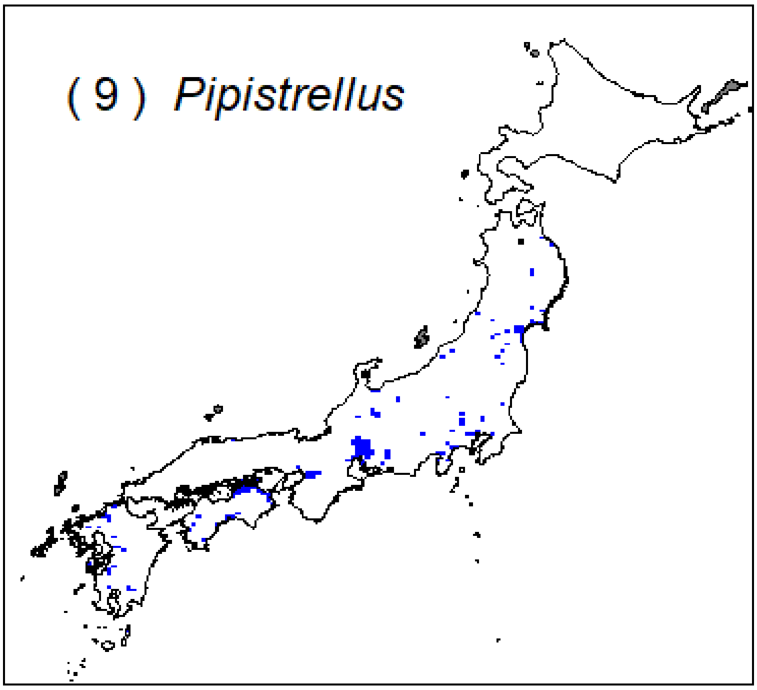
(9) *Pipistrellus*

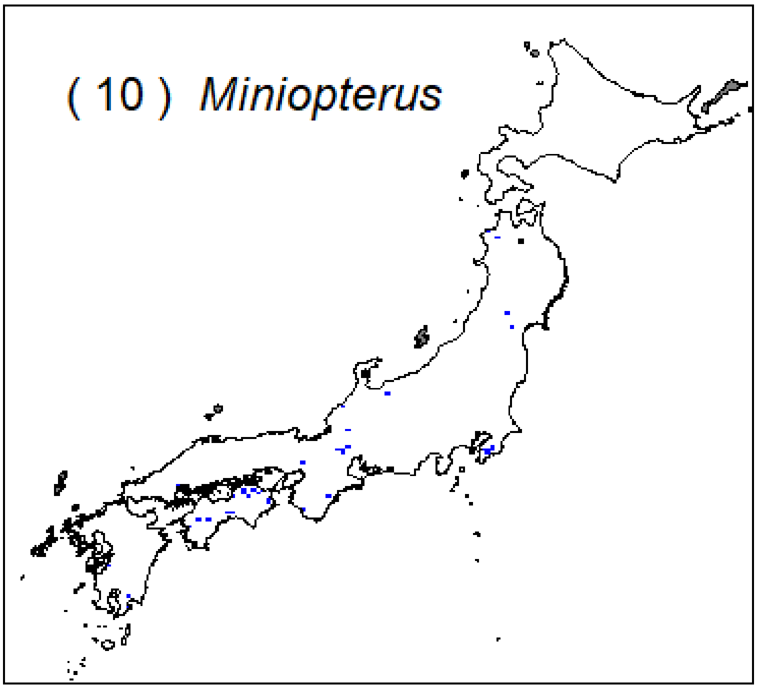
(10) *Miniopterus*

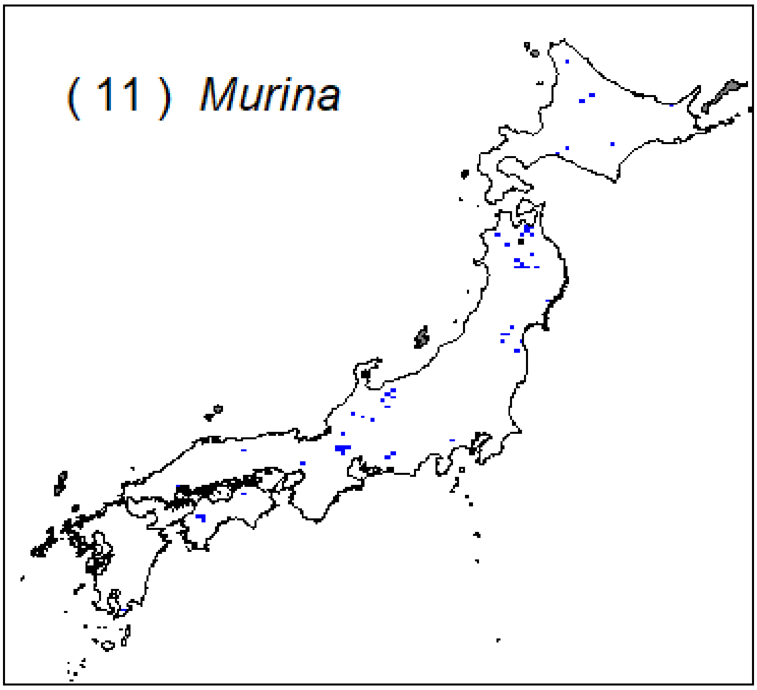
(11) Murina

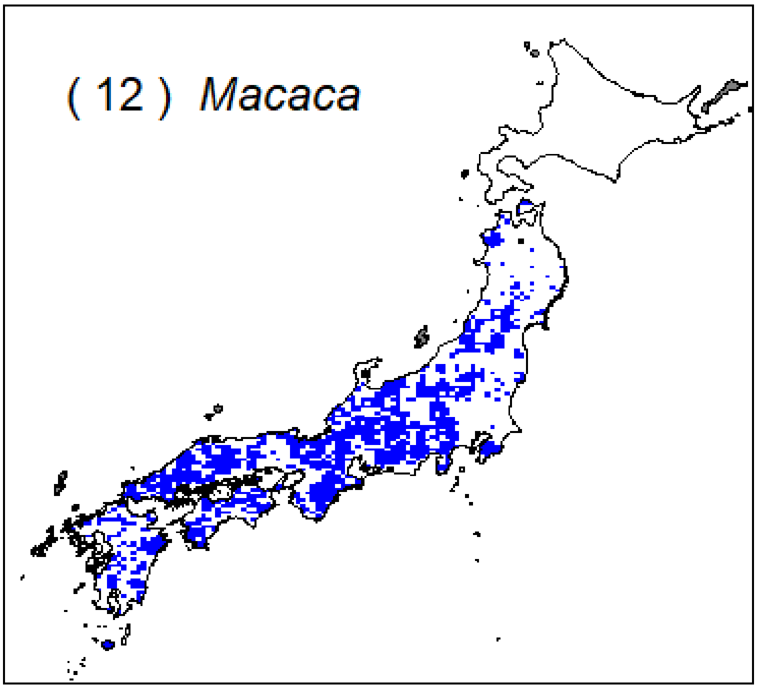
(12) Macaca

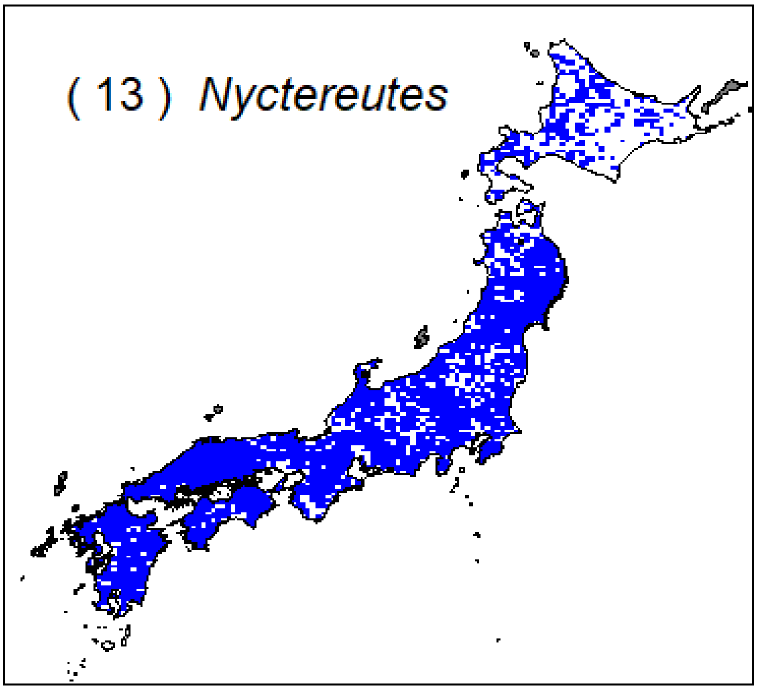
(13) *Nyctereutes*

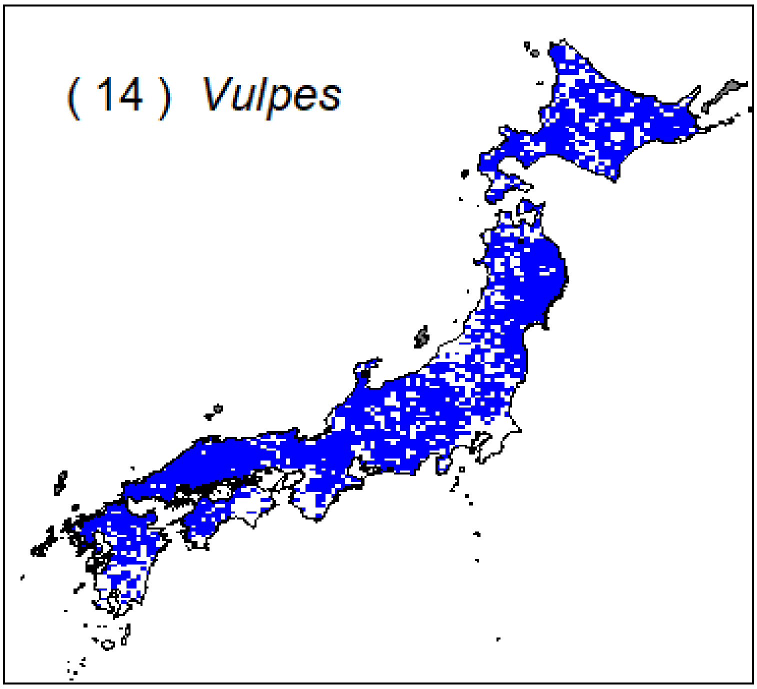
(14) *Vulpes*

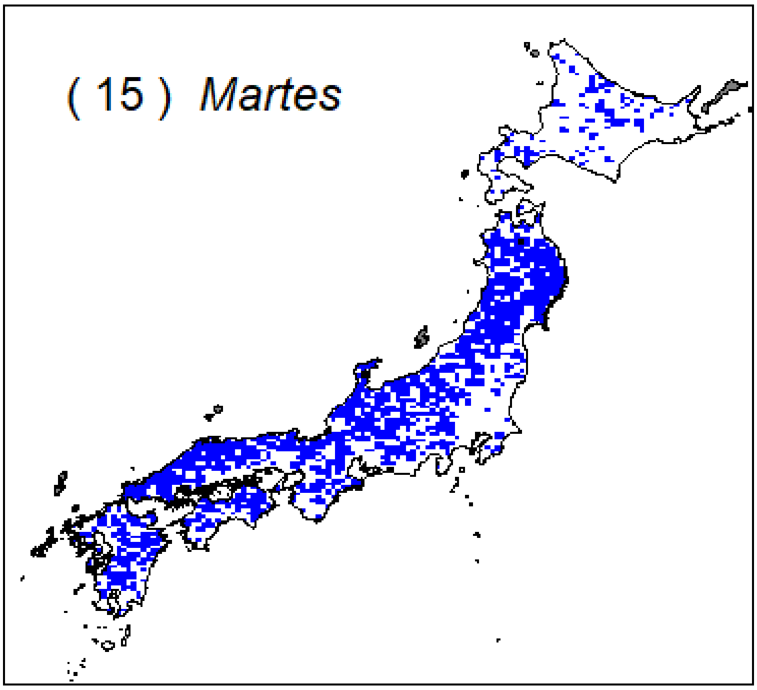
(15) *Martes*

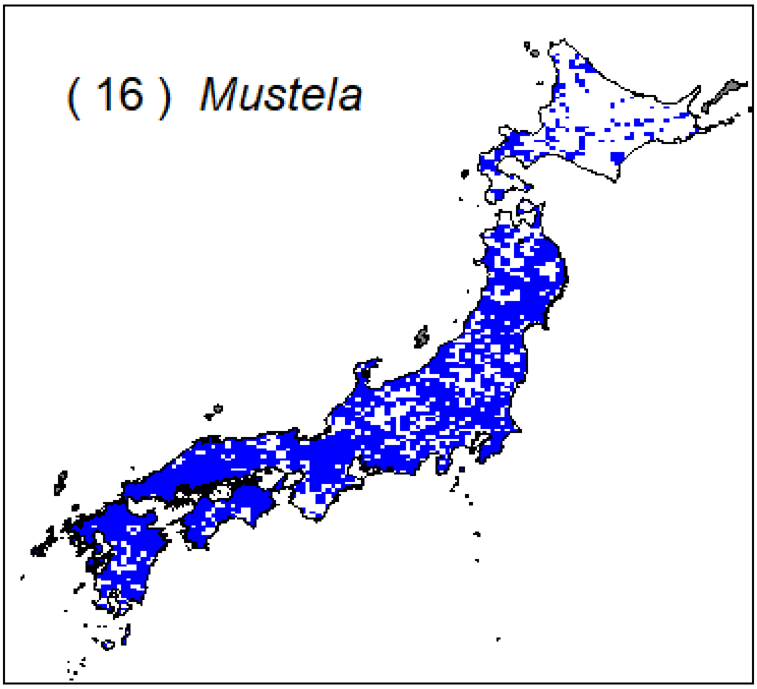
(16) *Mustela*

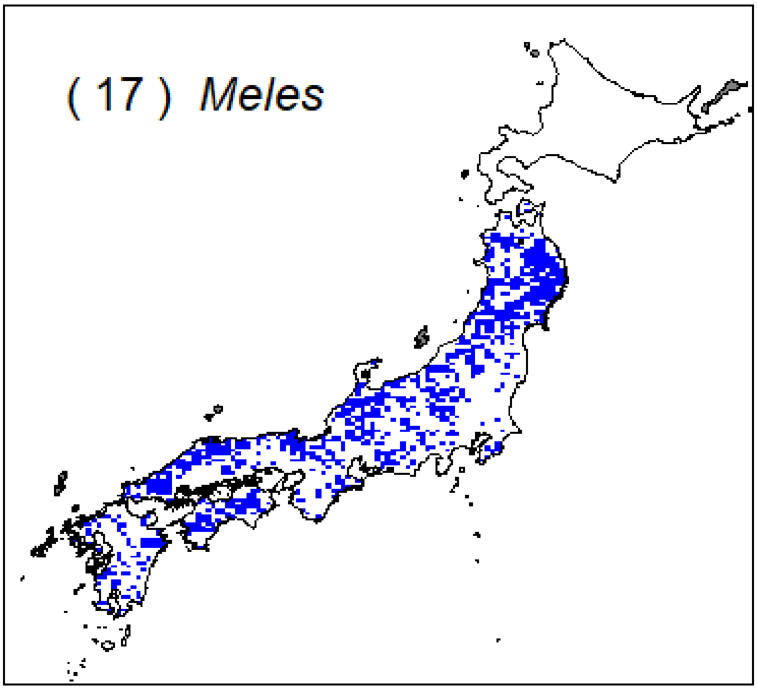
(17) *Meles*

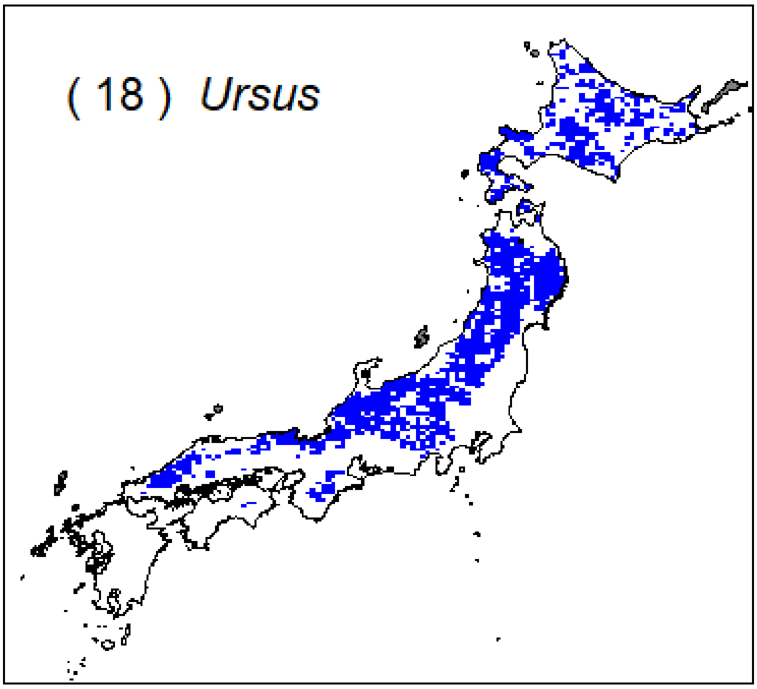
(18) *Ursus*

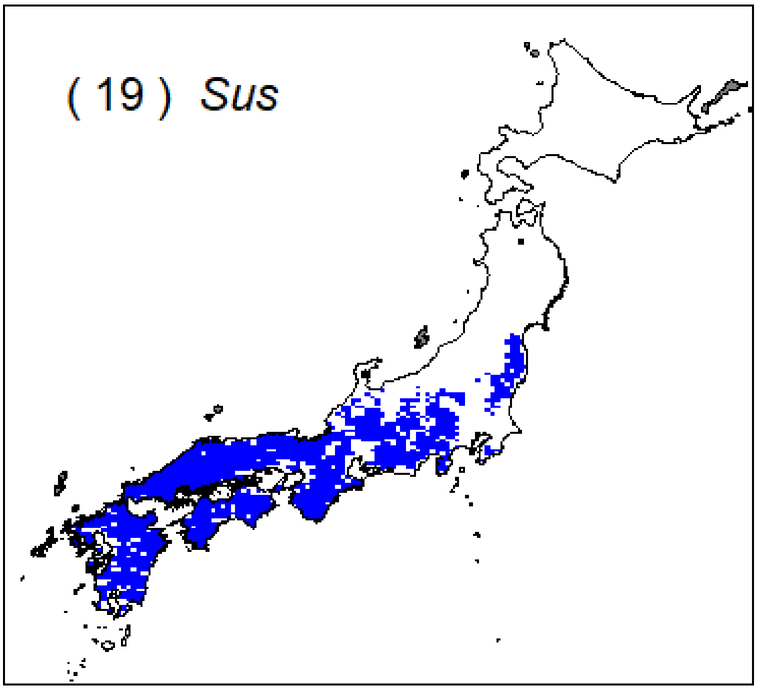
(19) *Sus*

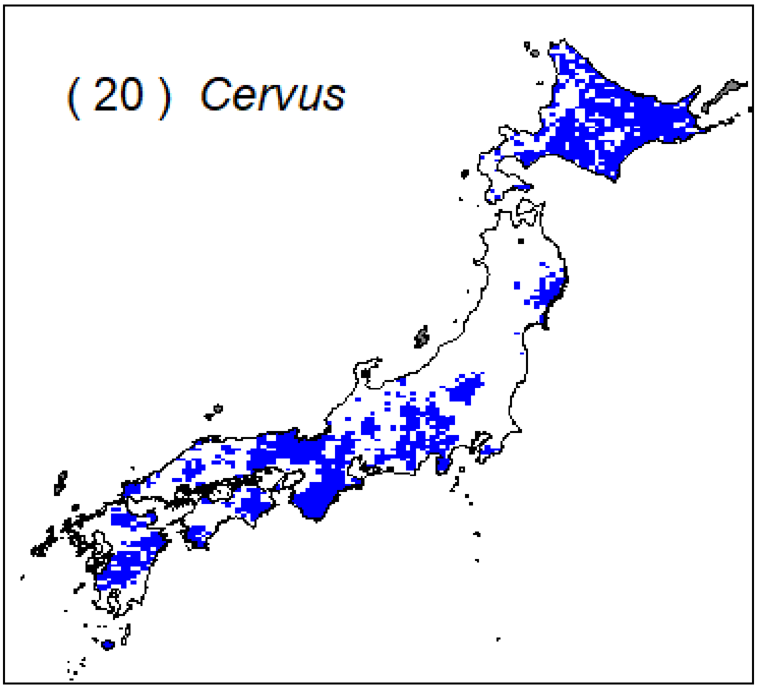
(20) *Cervus*

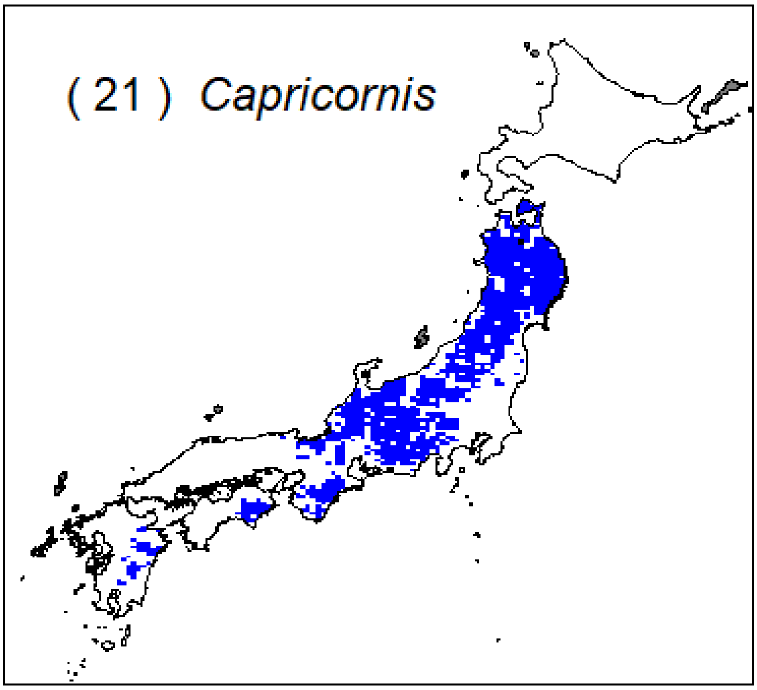
(21) *Capricornis*

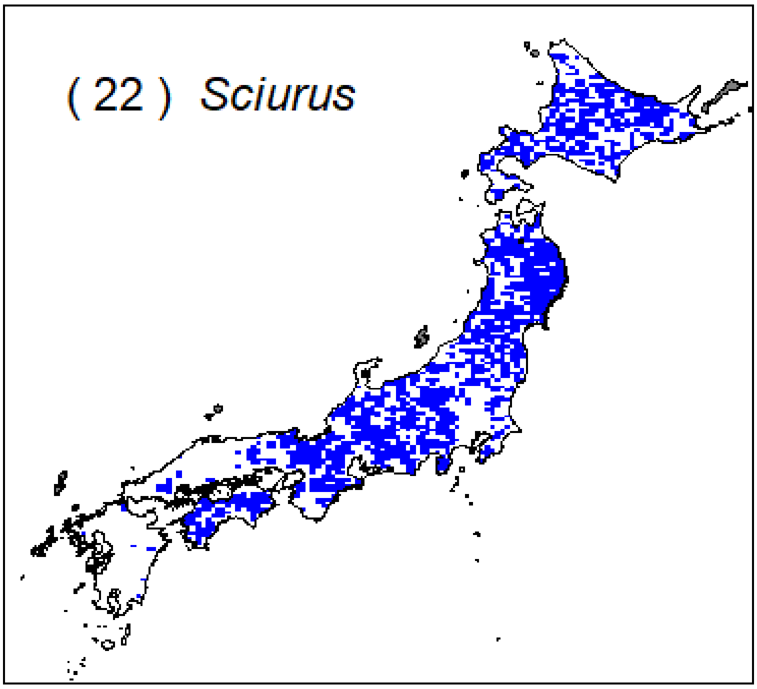
(22) *Sciurus*

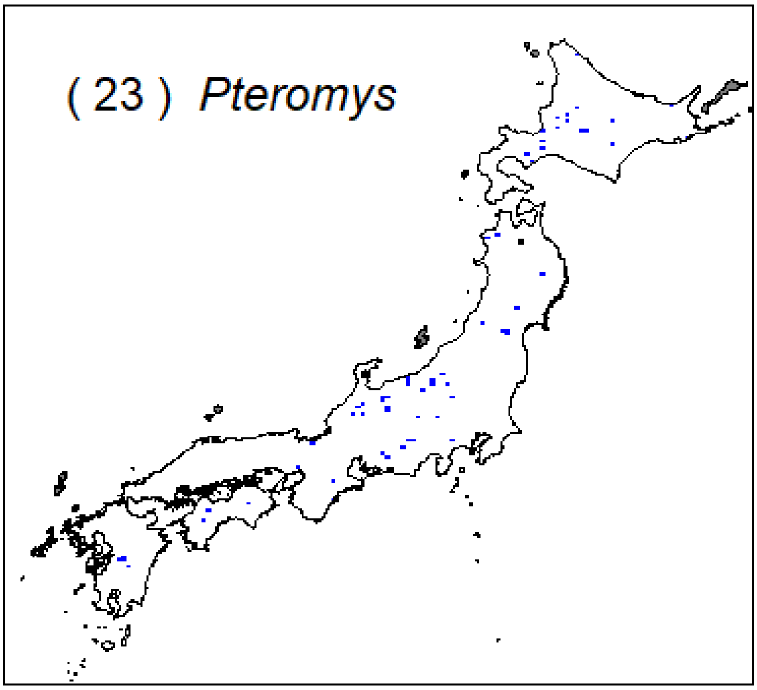
(23) Pteromys

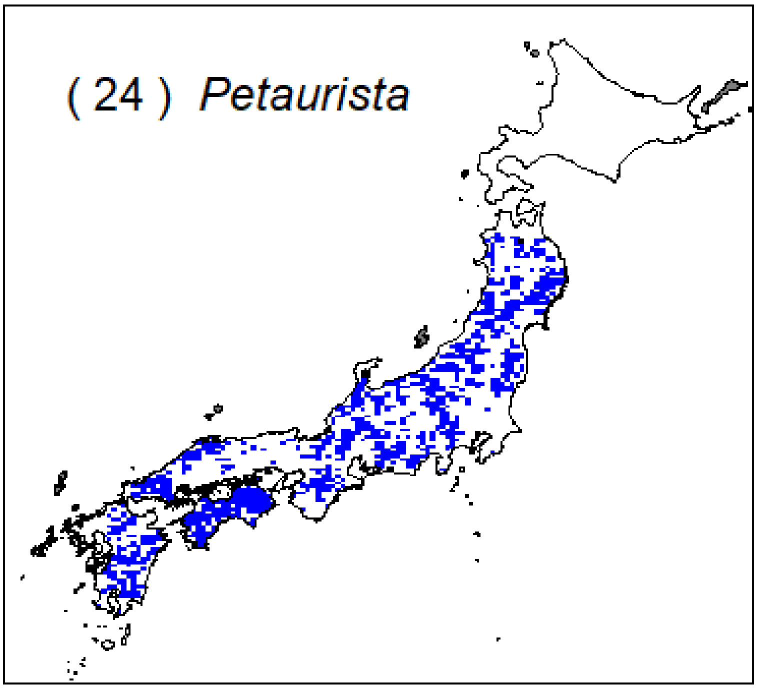
(24) Petaurista

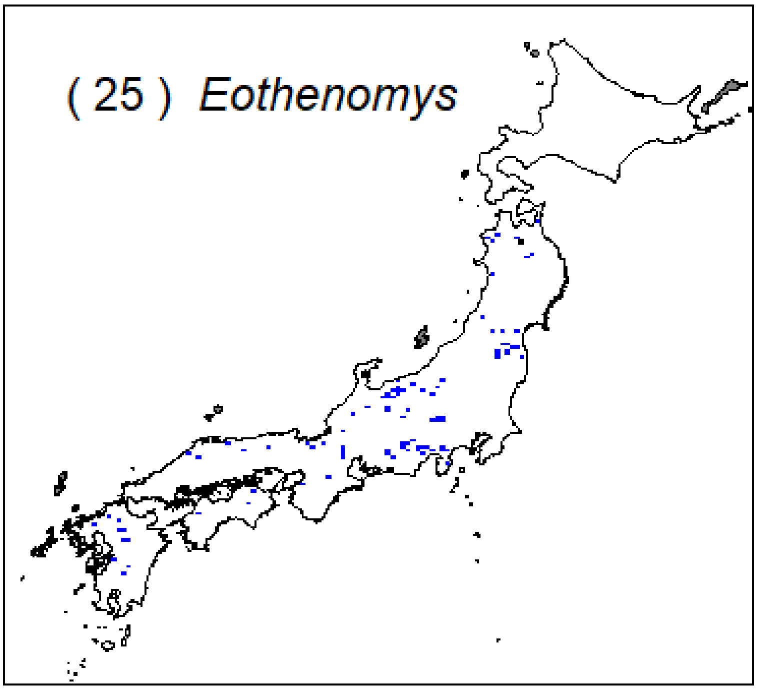
(25) *Eothenomys*

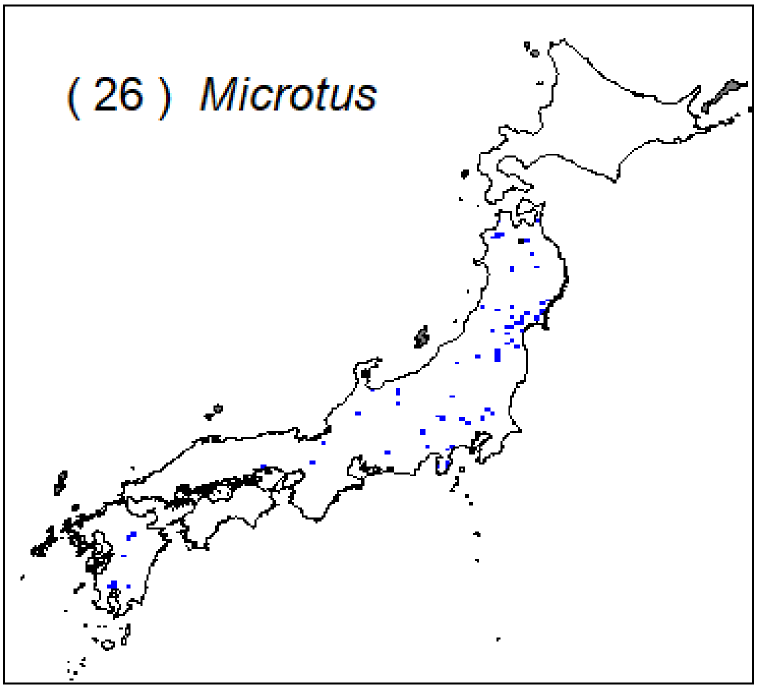
(26) *Microtus*

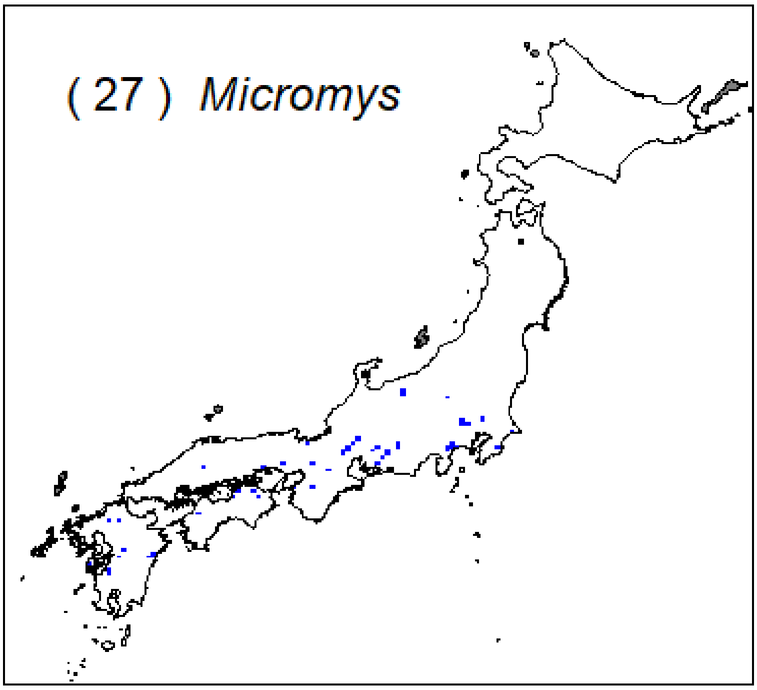
(27) Micromys

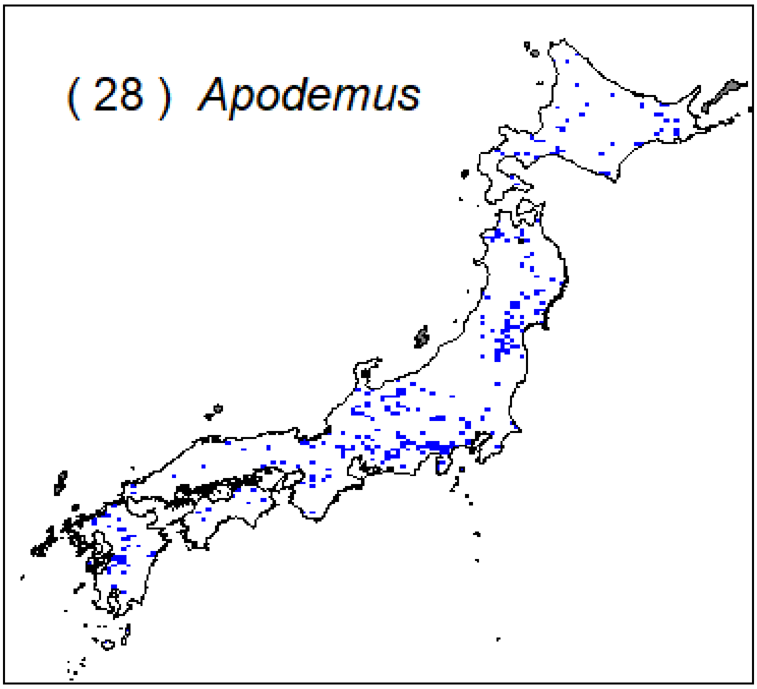
(28) Apodemus

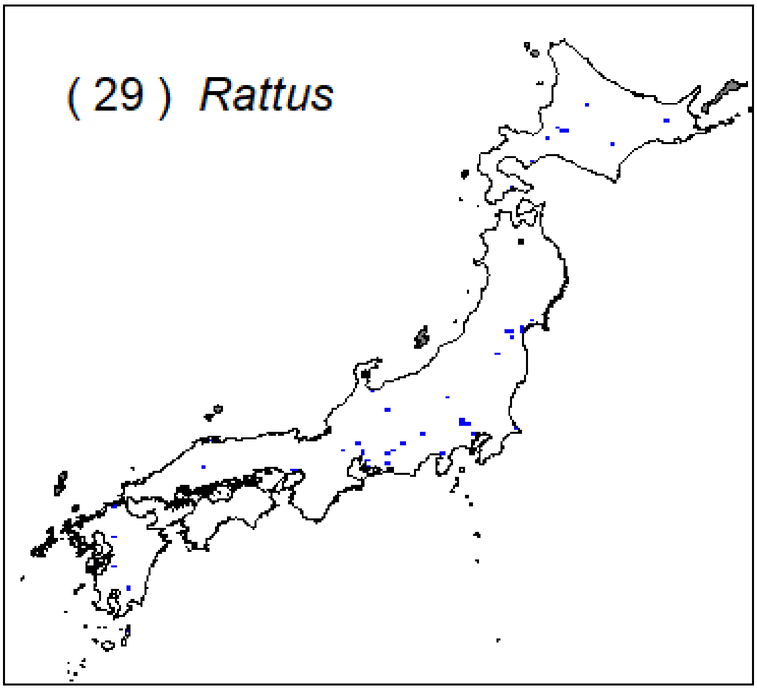
(29) *Rattus*

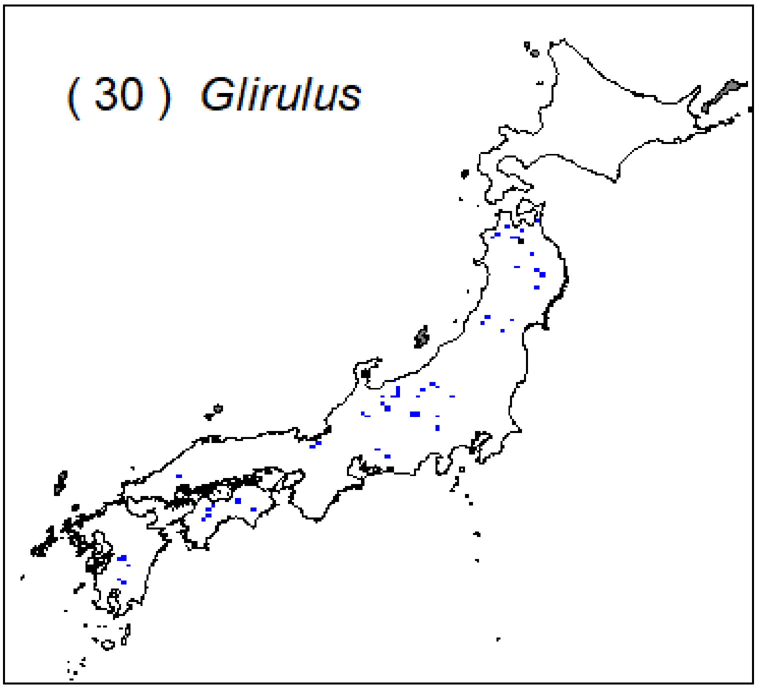
(30) *Glirulus*

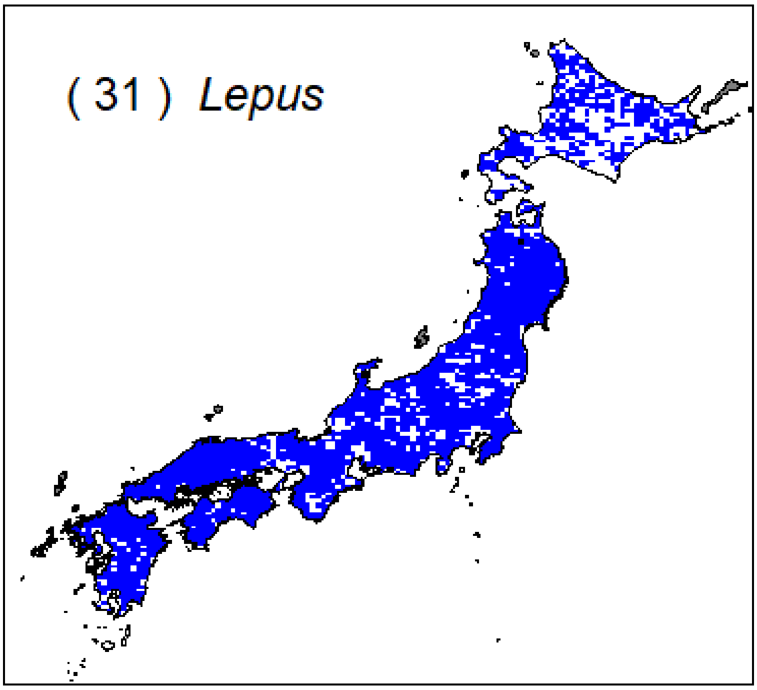
(31) *Lepus*

Supplementary Figure S2 Maps of settlement during six historical periods.

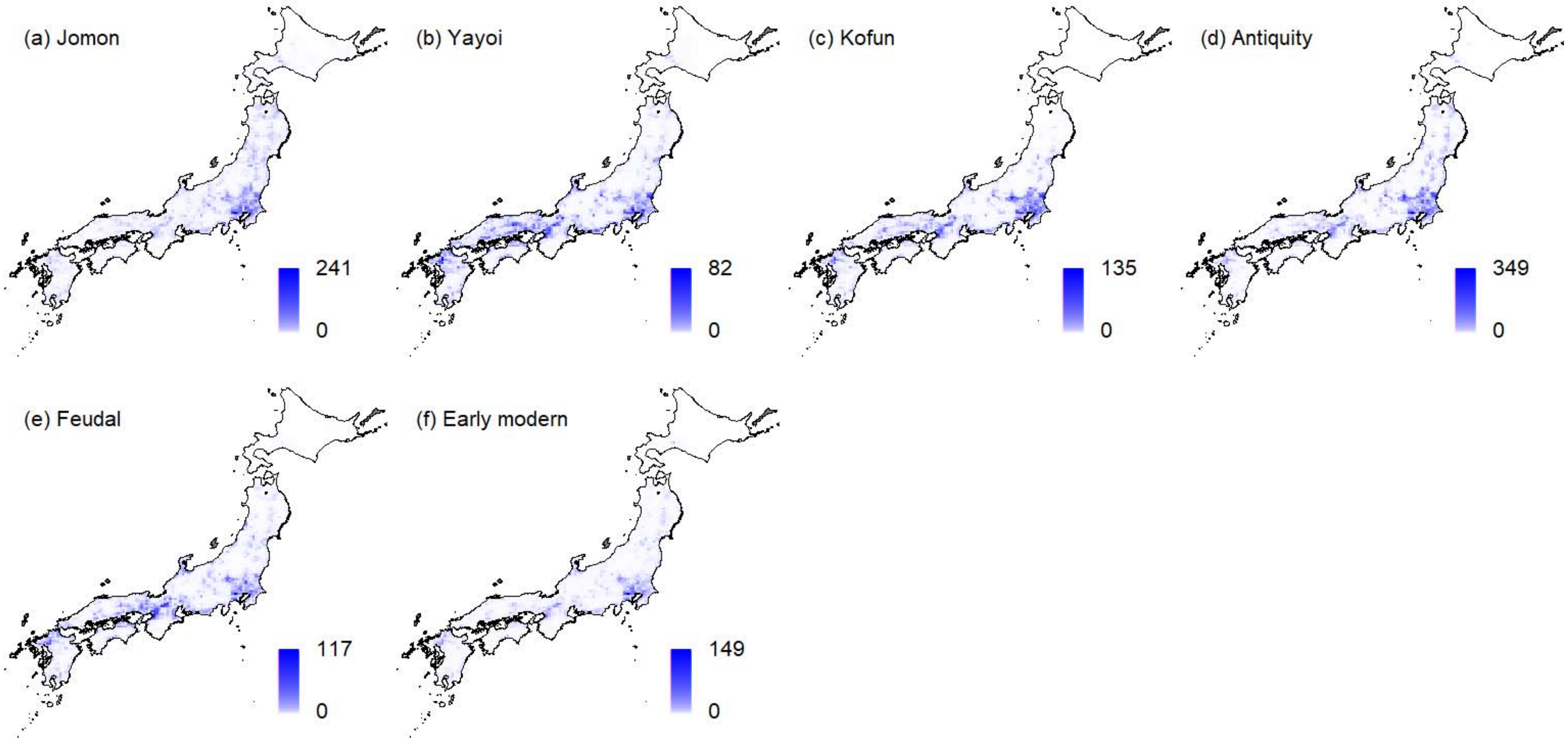

Supplementary Figure S3 Maps of ironwork during four historical periods.

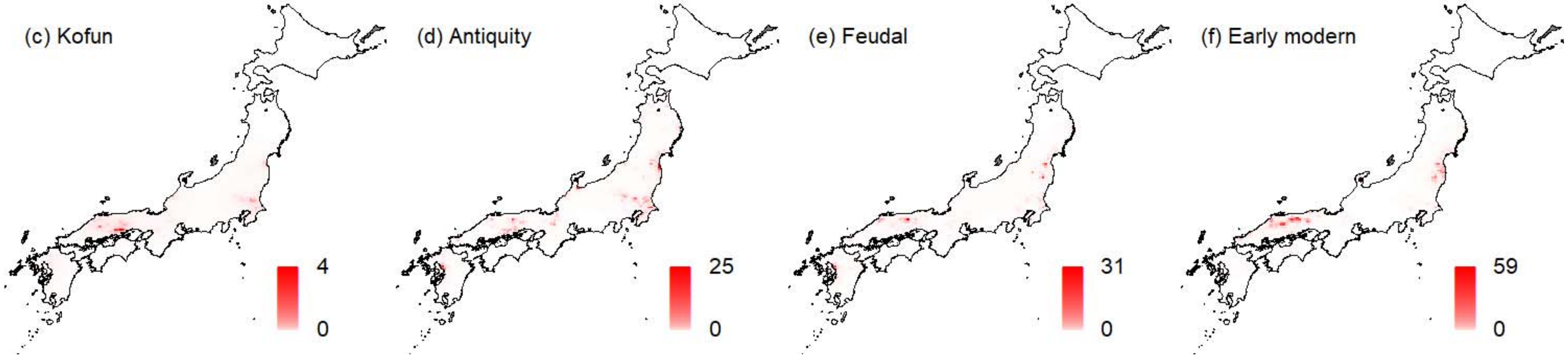

Supplementary Figure S4 Maps of kiln during four historical periods.

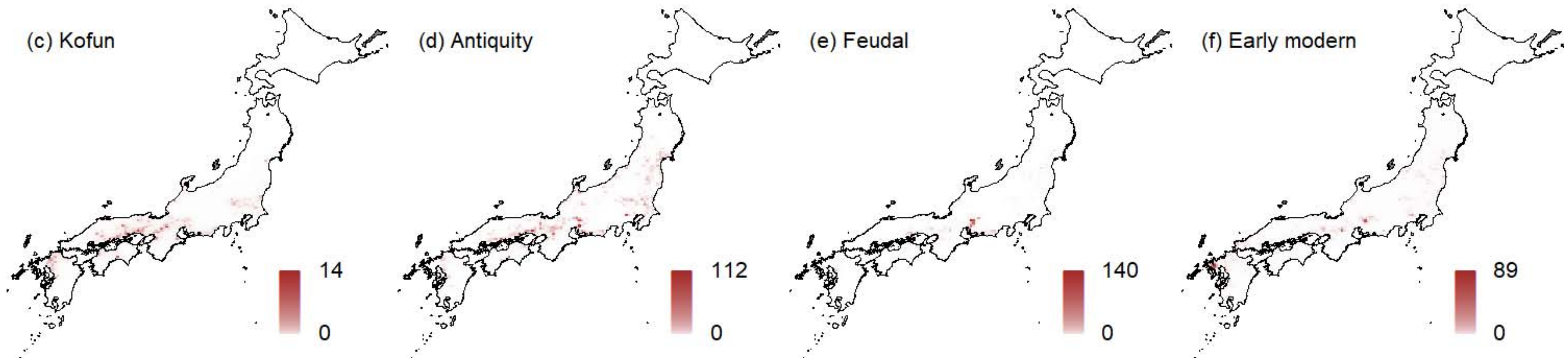

Supplementary Table S1 Traits and posterior mean (posterior standard deviation, lower and upper 95% credible limit) of the regression coefficients for each genus. Blue characters indicate negative effects (i.e. upper 95% limit is less than 0), and red characters indicate positive effect (i.e. lower 95% limit is larger than 0).

Family	Genus (common name)	Size class[*1]	ln(Relative dispersion of components of the fit)	Settlement						Ironwork				Kiln				Other factors													Intercept
				Early modern	Feudal	Antiquity	Kofun	Yayoi	Jomon	Early modern	Feudal	Antiquity	Kofun	Early modern	Feudal	Antiquity	Kofun	Mean annual temperture	Annual precipitation	Precipitation in summer	Maximum snow depth	Elevation	Topographic roughness	Current urban area	Current agricultural field	Mean annual temperture in the Mid-Holocene (difference from the current value)	Mean annual temperture in the Younger Dryas Stadial (difference from the Mid-Holocene)	Annual precipitation in the Mid-Holocene (difference from the current value)	Annual precipitation in the Younger Dryas Stadial (difference from the Mid-Holocene)	submerged land in the mid-Holocene transgression	
Soricomorpha	*Sorex* (shrew)	Small	-2.97	-0.0239 (0.0862, -0.202, 0.135)	-0.0212 (0.0846, -0.193, 0.139)	-0.0131 (0.106, -0.229, 0.188)	-0.00826 (0.128, -0.265, 0.237)	-0.00267 (0.0828, -0.17, 0.155)	-0.0314 (0.0907, -0.215, 0.14)	-0.0334 (0.0576, -0.163, 0.0635)	-0.024 (0.0594, -0.158, 0.0754)	-0.0146 (0.0523, -0.128, 0.0771)	-0.0184 (0.0554, -0.148, 0.0706)	-0.0235 (0.0568, -0.154, 0.0689)	-0.0161 (0.0563, -0.142, 0.0797)	-0.0161 (0.0571, -0.14, 0.0842)	-0.0115 (0.0537, -0.127, 0.084)	-0.29 (0.116, -0.508, -0.056)	-0.0701 (0.11, -0.287, 0.144)	0.0719 (0.109, -0.145, 0.285)	0.135 (0.0656, 0.00563, 0.263)	0.148 (0.0875, -0.0251, 0.319)	0.117 (0.075, -0.0314, 0.263)	0.0424 (0.0596, -0.0777, 0.156)	-0.00859 (0.0508, -0.109, 0.0902)	-0.114 (0.0478, -0.207, -0.0199)	-0.0667 (0.0515, -0.168, 0.0342)	0.00232 (0.0572, -0.11, 0.114)	0.00159 (0.0636, -0.122, 0.127)	0.122 (0.049, 0.0253, 0.217)	-1.98 (0.00454, -1.99, -1.97)
	Chimarrogale (water shrew)	Small																													
	Crocidura (shrew)	Small	0.991	-0.09 (0.199, -0.5, 0.278)	0.186 (0.179, -0.174, 0.536)	0.0379 (0.285, -0.545, 0.558)	-0.813 (0.417, -1.65, -0.0325)	0.431 (0.225, -0.013, 0.866)	-0.223 (0.248, -0.738, 0.229)	-1.71 (0.578, -2.83, -0.824)	-0.937 (0.464, -1.54, -0.408)	0.176 (0.129, -0.0684, 0.424)	-1.44 (1.01, -4.71, -0.702)	-0.156 (0.222, -0.728, 0.118)	-0.179 (0.162, -0.342, -0.0315)	-0.177 (0.196, -0.62, 0.128)	-0.0397 (0.144, -0.365, 0.191)	1.51 (0.623, 0.265, 2.86)	-0.574 (0.461, -1.5, 0.311)	0.584 (0.532, -0.438, 1.65)	-0.0265 (0.289, -0.603, 0.536)	0.196 (0.419, -0.62, 1.03)	0.758 (0.178, 0.412, 1.11)	0.426 (0.126, 0.178, 0.671)	0.105 (0.13, -0.159, 0.373)	0.282 (0.176, -0.0574, 0.632)	0.213 (0.325, -0.436, 0.839)	0.000927 (0.176, -0.35, 0.348)	0.147 (0.229, -0.29, 0.614)	-0.208 (0.149, -0.504, 0.0798)	-7.83 (0.871, -9.61, -6.1)
	Dymecodon (shrew-mole)	Small																													
	Urotrichus (shrew-mole)	Small	-3.76	0.405 (0.162, 0.0945, 0.732)	-0.592 (0.218, -1.05, -0.189)	0.000897 (0.226, -0.462, 0.417)	-0.0255 (0.263, -0.552, 0.477)	0.149 (0.176, -0.193, 0.495)	-0.102 (0.176, -0.455, 0.234)	-0.027 (0.0836, -0.212, 0.113)	-0.0975 (0.107, -0.349, 0.0673)	0.163 (0.0653, 0.0338, 0.291)	-0.582 (0.576, -2.22, -0.121)	-0.392 (0.265, -1.02, -0.0241)	0.0204 (0.0623, -0.111, 0.139)	-0.0323 (0.0655, -0.181, 0.0886)	0.143 (0.0662, 0.00386, 0.266)	-4.4 (1.32, -7.15, -1.97)	-0.749 (0.406, -1.57, 0.0323)	0.91 (0.456, 0.0279, 1.82)	0.11 (0.221, -0.333, 0.564)	-2.41 (0.682, -3.83, -1.15)	0.637 (0.142, 0.361, 0.919)	0.203 (0.132, -0.0602, 0.457)	-0.22 (0.142, -0.504, 0.0521)	-0.151 (0.154, -0.459, 0.147)	0.352 (0.483, -0.611, 1.28)	0.000683 (0.169, -0.334, 0.331)	1.82 (0.46, 0.948, 2.77)	-0.266 (0.143, -0.552, 0.0113)	-3.85 (0.848, -5.58, -2.23)
	Mogera (mole)	Small	-1.75	0.0952 (0.102, -0.105, 0.297)	-0.286 (0.133, -0.557, -0.0343)	0.043 (0.131, -0.221, 0.298)	0.101 (0.174, -0.242, 0.44)	-0.00816 (0.12, -0.244, 0.226)	-0.00919 (0.114, -0.235, 0.211)	-0.232 (0.191, -0.671, 0.0557)	-0.195 (0.168, -0.583, 0.0636)	0.0502 (0.0667, -0.0862, 0.177)	-0.154 (0.141, -0.485, 0.0518)	-0.125 (0.0918, -0.335, 0.0232)	0.0763 (0.0523, -0.0242, 0.183)	-0.145 (0.0832, -0.335, -0.00482)	0.0155 (0.0718, -0.14, 0.142)	-0.541 (0.725, -1.98, 0.873)	-0.0375 (0.374, -0.776, 0.696)	0.739 (0.43, -0.0983, 1.59)	-0.097 (0.236, -0.564, 0.363)	-0.409 (0.412, -1.22, 0.394)	0.435 (0.138, 0.163, 0.707)	0.381 (0.0979, 0.19, 0.575)	0.316 (0.0997, 0.122, 0.513)	0.269 (0.155, -0.0341, 0.575)	-0.456 (0.343, -1.14, 0.209)	0.358 (0.151, 0.0644, 0.657)	0.177 (0.228, -0.261, 0.632)	0.0873 (0.0983, -0.105, 0.281)	-3.35 (0.822, -5.01, -1.77)
Chiroptera	*Rhinolophus* (horseshoe bat)	Small	-0.619	-0.00537 (0.239, -0.498, 0.427)	0.345 (0.251, -0.147, 0.839)	-0.466 (0.457, -1.44, 0.325)	-1.51 (0.635, -2.85, -0.361)	0.661 (0.292, 0.0773, 1.22)	0.148 (0.287, -0.439, 0.684)	0.124 (0.0878, -0.0585, 0.289)	0.0541 (0.061, -0.0687, 0.172)	0.089 (0.0734, -0.0639, 0.226)	-0.686 (0.892, -3.54, -0.231)	-0.0876 (0.0917, -0.297, 0.061)	0.124 (0.071, -0.0046, 0.276)	-0.0435 (0.0969, -0.255, 0.125)	-0.000295 (0.123, -0.274, 0.205)	1.22 (0.915, -0.502, 3.11)	-0.685 (0.612, -1.98, 0.427)	0.984 (0.693, -0.253, 2.49)	0.0433 (0.343, -0.63, 0.721)	0.0182 (0.548, -1.04, 1.11)	0.863 (0.229, 0.474, 1.4)	0.162 (0.182, -0.2, 0.518)	-0.00935 (0.174, -0.357, 0.328)	0.613 (0.244, 0.199, 1.18)	0.32 (0.499, -0.649, 1.31)	0.293 (0.229, -0.138, 0.767)	1.1 (0.437, 0.335, 2.06)	-0.291 (0.165, -0.63, 0.0187)	-8.97 (1.36, -11.8, -6.76)
	Myotis (myotis)	Small	-1.43	0.282 (0.4, -0.658, 0.868)	-0.612 (0.524, -1.72, 0.373)	0.851 (0.617, -0.407, 1.98)	-1.59 (1.25, -4.24, 0.56)	0.97 (0.511, -0.0617, 2.01)	-0.814 (0.859, -2.94, 0.0587)	-0.713 (0.234, -1.08, -0.431)	-0.159 (0.312, -0.79, 0.269)	0.187 (0.154, -0.0695, 0.47)	-0.344 (0.334, -1.18, 0.0733)	-1.03 (0.968, -3.35, 0.0504)	0.541 (0.199, 0.242, 0.99)	-0.195 (0.212, -0.69, 0.135)	0.174 (0.151, -0.148, 0.433)	2.36 (1.66, -1.35, 4.96)	-3.69 (1.79, -7.94, -0.651)	3.67 (1.83, 0.446, 7.77)	1.25 (0.747, -0.113, 2.88)	1.4 (1.03, -0.815, 3.11)	1.38 (0.516, 0.558, 2.64)	-0.0149 (0.432, -0.984, 0.713)	0.624 (0.382, -0.0959, 1.43)	0.552 (0.467, -0.282, 1.58)	0.576 (0.784, -0.992, 2.07)	-0.555 (0.563, -1.75, 0.488)	1.12 (0.813, -0.424, 2.74)	-0.211 (0.371, -0.969, 0.496)	-11.1 (1.81, -13.6, -8.62)
	Pipistrellus (pipistrelle)	Small	-0.394	0.312 (0.201, -0.0521, 0.765)	-0.17 (0.214, -0.654, 0.231)	-0.683 (0.415, -1.73, -0.0935)	0.82 (0.416, 0.135, 1.82)	-0.173 (0.224, -0.68, 0.225)	-0.0899 (0.222, -0.562, 0.326)	-0.399 (0.275, -0.963, 0.117)	-0.263 (0.242, -0.681, 0.146)	-0.0778 (0.162, -0.433, 0.192)	-1.42 (1.46, -5.59, -0.651)	-1.36 (0.71, -3.04, -0.341)	0.57 (0.263, 0.225, 1.25)	-0.0357 (0.117, -0.272, 0.184)	-0.419 (0.253, -1.05, -0.0544)	0.964 (2.08, -3.54, 4.48)	-4.49 (1.68, -8.54, -1.74)	3.25 (1.61, 0.521, 7.01)	0.638 (0.769, -0.926, 2.1)	-1.65 (1.48, -4.91, 0.928)	1.22 (0.496, 0.486, 2.52)	1.32 (0.386, 0.738, 2.35)	0.578 (0.285, 0.144, 1.3)	-0.0602 (0.373, -0.807, 0.667)	0.108 (0.696, -1.27, 1.47)	-0.615 (0.509, -1.72, 0.304)	0.273 (0.466, -0.611, 1.23)	-0.0417 (0.205, -0.458, 0.36)	-13.1 (3.28, -19.2, -7.1)
	Miniopterus (long-winged bat)	Small	0.472	-0.366 (1.05, -2.79, 0.813)	1.55 (0.766, -0.232, 2.87)	0.152 (1.84, -4.25, 2.61)	-5.21 (3.08, -10.6, -0.654)	0.331 (0.945, -1.77, 1.89)	0.516 (1.13, -3.62, 1.3)	-3.57 (2.01, -6.61, -0.761)	0.493 (0.262, 0.0443, 1.07)	-0.322 (0.357, -1.04, 0.218)	-0.157 (0.33, -0.93, 0.277)	-1.36 (0.202, -1.73, -0.936)	-1.1 (0.217, -1.51, -0.669)	-0.0322 (0.291, -0.518, 0.41)	0.462 (0.154, 0.127, 0.731)	-2.16 (3.53, -9.18, 4.52)	-1.55 (2.15, -6.04, 2.44)	0.416 (2.4, -4.09, 5.45)	-0.476 (1.3, -3.16, 1.94)	-5.53 (2.61, -10.9, -0.699)	3.52 (1.09, 1.56, 5.55)	-0.177 (0.77, -1.95, 1.06)	0.238 (0.597, -0.965, 1.39)	2.97 (0.944, 1.3, 5.12)	0.723 (1.36, -1.99, 3.18)	-0.511 (0.887, -2.25, 1.23)	0.955 (0.967, -0.866, 2.86)	-0.108 (0.485, -1.15, 0.84)	-26.9 (1.93, -30, -22.2)
	Murina (tube-nosed bat)	Small	0.502	-2.86 (2.54, -9.19, 0.0891)	-0.0235 (0.805, -1.09, 1.76)	-0.305 (1.49, -3.01, 1.42)	0.552 (1.54, -3.94, 1.9)	-1.23 (1.16, -2.73, 0.93)	0.0583 (1.08, -2.36, 1.42)	0.163 (0.153, -0.176, 0.436)	-0.104 (0.183, -0.54, 0.175)	0.352 (0.147, 0.113, 0.597)	-1.75 (0.38, -2.53, -1.19)	-2.03 (0.433, -2.99, -1.3)	-9.55 (1.46, -12.3, -7.14)	-0.487 (0.548, -1.72, 0.372)	-0.736 (0.259, -1.18, -0.37)	-2.39 (3.54, -9.66, 4.04)	-11.5 (3.45, -19.1, -5.85)	9.14 (2.68, 4.52, 14.9)	2.84 (1.09, 1.07, 5.09)	-1.43 (1.81, -5.44, 1.62)	1.56 (0.54, 0.522, 2.66)	0.834 (0.439, -0.194, 1.51)	-0.13 (0.513, -1.19, 0.874)	1.92 (0.836, 0.608, 3.88)	0.769 (1.15, -1.64, 2.8)	-1.72 (0.921, -3.72, -0.0571)	1.63 (1.74, -1.03, 4.71)	-1.14 (0.759, -2.84, 0.0682)	-21.6 (1.81, -26.2, -18.8)
Primates	*Macaca* (macaque)	Medium	-4.71	-0.0429 (0.111, -0.269, 0.169)	-0.109 (0.105, -0.32, 0.0963)	0.0224 (0.142, -0.263, 0.298)	-0.189 (0.173, -0.535, 0.147)	0.288 (0.11, 0.0774, 0.51)	0.026 (0.123, -0.218, 0.267)	0.00558 (0.0493, -0.0922, 0.103)	0.0703 (0.0515, -0.0281, 0.181)	-0.0913 (0.0679, -0.236, 0.0314)	-0.0917 (0.0573, -0.218, 0.00831)	-0.0651 (0.0567, -0.184, 0.0387)	0.0477 (0.053, -0.0527, 0.157)	0.0257 (0.0487, -0.0735, 0.121)	-0.0802 (0.0532, -0.195, 0.0179)	-3.13 (0.799, -4.75, -1.61)	-0.116 (0.322, -0.748, 0.518)	0.722 (0.355, 0.0299, 1.42)	-0.215 (0.184, -0.578, 0.146)	-2.32 (0.437, -3.2, -1.49)	1.07 (0.112, 0.856, 1.3)	-0.14 (0.1, -0.339, 0.0536)	-0.149 (0.0893, -0.326, 0.0252)	0.29 (0.122, 0.0499, 0.53)	0.734 (0.242, 0.265, 1.21)	-0.0652 (0.133, -0.326, 0.197)	0.592 (0.173, 0.258, 0.936)	0.121 (0.0741, -0.0238, 0.267)	-0.289 (0.81, -1.87, 1.31)
Rodentia	*Sciurus* (squirrel)	Medium	-5.12	-0.269 (0.116, -0.506, -0.0495)	0.131 (0.105, -0.074, 0.34)	0.197 (0.127, -0.0493, 0.45)	-0.347 (0.161, -0.669, -0.0379)	0.0868 (0.109, -0.128, 0.301)	0.19 (0.111, -0.0281, 0.409)	-0.0506 (0.0824, -0.227, 0.0924)	0.108 (0.0597, -0.00259, 0.232)	0.0297 (0.0493, -0.067, 0.127)	-0.0579 (0.0788, -0.238, 0.0688)	0.0416 (0.0527, -0.0652, 0.142)	-0.117 (0.0621, -0.248, -0.00313)	0.0101 (0.0505, -0.0935, 0.106)	0.00776 (0.0522, -0.0968, 0.109)	-3.14 (0.795, -4.77, -1.66)	-0.537 (0.301, -1.13, 0.0509)	0.271 (0.325, -0.367, 0.909)	-0.385 (0.145, -0.671, -0.102)	-2.48 (0.423, -3.34, -1.68)	0.988 (0.102, 0.79, 1.19)	-0.296 (0.095, -0.485, -0.112)	0.0302 (0.0679, -0.103, 0.163)	0.355 (0.11, 0.139, 0.57)	0.454 (0.178, 0.105, 0.805)	-0.214 (0.122, -0.456, 0.025)	0.8 (0.175, 0.462, 1.15)	0.00888 (0.0663, -0.121, 0.14)	-1.29 (0.106, -1.5, -1.09)
	Pteromys (dwarf flying squirrel)	Medium	3.27	-4.32 (3.97, -13.7, 0.761)	-3.79 (0.905, -5.71, -2.18)	-0.0579 (0.944, -1.98, 1.11)	-2.23 (3.51, -15.1, 0.279)	-2.92 (2.57, -5.53, 1.62)	-1.96 (2.55, -8.6, 0.411)	-12.1 (1.9, -16.1, -8.63)	-10 (2.03, -14.4, -6.61)	-3.7 (0.595, -4.87, -2.53)	-7.57 (3.74, -12.9, -3.47)	-6.05 (0.78, -7.62, -4.55)	-10.6 (1.65, -14.1, -7.6)	-6.06 (0.8, -7.78, -4.63)	-2.8 (0.641, -4.14, -1.63)	-5.76 (2.76, -11.2, -0.435)	-0.98 (1.2, -3.82, 0.545)	1.24 (1.23, -1.1, 3.75)	0.28 (0.494, -0.648, 1.46)	-1.42 (1.35, -4.52, 0.774)	1.04 (0.537, 0.457, 2.06)	1.94 (0.366, 1.2, 2.62)	0.559 (0.309, 0.000833, 1.22)	0.591 (0.384, -0.0286, 1.49)	-0.235 (0.959, -2.21, 1.44)	0.211 (0.363, -0.661, 1.02)	1.22 (0.903, -0.338, 3.36)	-0.146 (0.422, -0.996, 0.666)	-42 (2.42, -44.8, -35.6)
	Petaurista (giant flying squirrel)	Medium	-5.17	0.142 (0.112, -0.0726, 0.381)	0.0855 (0.125, -0.162, 0.329)	-0.0847 (0.155, -0.399, 0.217)	-0.423 (0.197, -0.825, -0.0503)	0.0491 (0.13, -0.207, 0.302)	0.254 (0.132, -0.00756, 0.511)	-0.0933 (0.0692, -0.239, 0.0314)	0.104 (0.0504, 0.0101, 0.212)	-0.0444 (0.0616, -0.173, 0.0701)	-0.247 (0.19, -0.707, 0.0166)	-0.21 (0.143, -0.534, 0.0129)	-0.0706 (0.0781, -0.251, 0.0601)	0.00143 (0.0592, -0.122, 0.113)	0.0776 (0.051, -0.0242, 0.18)	-7.17 (1.14, -9.48, -5)	0.0189 (0.349, -0.667, 0.706)	0.809 (0.376, 0.0749, 1.55)	0.000616 (0.187, -0.366, 0.367)	-4.43 (0.597, -5.64, -3.29)	0.903 (0.112, 0.689, 1.13)	-0.0269 (0.115, -0.257, 0.196)	-0.237 (0.0958, -0.427, -0.0505)	0.16 (0.129, -0.0937, 0.414)	0.809 (0.285, 0.253, 1.37)	0.499 (0.146, 0.215, 0.789)	1.05 (0.226, 0.615, 1.5)	0.0336 (0.0854, -0.133, 0.203)	-1.71 (0.943, -3.58, 0.125)
	Eothenomys (vole)	Small	-1.04	-0.357 (0.431, -1.09, 0.484)	0.0852 (0.471, -0.964, 0.946)	0.643 (0.58, -0.538, 1.75)	-1.84 (1.29, -4.97, 0.174)	-0.133 (0.605, -1.45, 0.917)	-0.153 (0.47, -1.08, 0.737)	-0.118 (0.15, -0.48, 0.108)	0.0385 (0.0881, -0.155, 0.192)	0.3 (0.122, 0.105, 0.597)	-2.59 (1.7, -6.82, -0.24)	-0.668 (0.75, -2.86, -0.0523)	-1.29 (1.08, -3.74, 0.0234)	-0.223 (0.361, -1.06, 0.214)	-0.0107 (0.233, -0.514, 0.347)	-4.59 (3.84, -15.2, 0.00191)	-1.95 (1.43, -6.03, -0.294)	1.81 (1.4, 0.141, 5.81)	0.445 (0.521, -0.345, 1.82)	-2.3 (1.87, -7.48, -0.0153)	1.06 (0.492, 0.484, 2.43)	0.431 (0.296, -0.163, 1.04)	-0.5 (0.373, -1.39, 0.124)	-0.172 (0.254, -0.761, 0.276)	0.146 (0.896, -1.74, 1.9)	-0.304 (0.358, -1.21, 0.28)	1.73 (1.05, 0.0619, 4.32)	-1.14 (0.646, -2.92, -0.251)	-8.69 (3.95, -19.7, -4.8)
	Microtus (vole)	Small	-2.87	0.62 (0.358, 0.0569, 1.52)	-0.066 (0.344, -0.82, 0.541)	-0.26 (0.375, -1.11, 0.404)	0.22 (0.374, -0.525, 1.02)	-0.185 (0.325, -0.921, 0.411)	-0.74 (0.421, -1.8, -0.104)	-0.339 (0.301, -0.951, 0.0707)	-0.128 (0.278, -0.661, 0.228)	0.0304 (0.119, -0.211, 0.267)	0.0298 (0.129, -0.241, 0.223)	-0.361 (0.453, -1.49, 0.17)	-2.18 (1.73, -7.49, -0.276)	0.24 (0.138, -0.0495, 0.496)	-0.371 (0.42, -1.37, 0.17)	-10.1 (6, -24.4, -1.73)	-2.68 (1.66, -7.34, -0.498)	2.61 (1.58, 0.414, 6.86)	-0.304 (0.588, -1.59, 0.771)	-5.02 (2.95, -11.8, -0.937)	0.888 (0.419, 0.29, 1.98)	0.426 (0.304, -0.107, 1.08)	0.516 (0.317, 0.0317, 1.32)	-0.868 (0.588, -2.23, 0.0117)	-0.206 (0.816, -1.92, 1.39)	-0.661 (0.55, -1.97, 0.183)	1.05 (0.928, -0.532, 3.08)	0.513 (0.341, -0.0155, 1.35)	-8.57 (3.1, -14.7, -3.99)
	Micromys (vole)	Small	1.42	0.338 (0.345, -0.37, 1.02)	-0.177 (0.355, -0.903, 0.507)	-1.47 (0.875, -3.41, -0.121)	1.99 (0.715, 0.654, 3.4)	-0.0367 (0.351, -0.773, 0.633)	-1.19 (0.563, -2.34, -0.333)	-12.6 (1.53, -15.8, -9.78)	-7.47 (2.44, -12.1, -3.81)	-0.0709 (0.385, -0.94, 0.45)	-0.717 (0.213, -1.12, -0.41)	-0.438 (0.375, -1.32, 0.0293)	0.186 (0.127, -0.0382, 0.457)	-0.366 (0.353, -1.21, 0.091)	-0.208 (0.249, -0.739, 0.147)	-7.82 (3.85, -15.1, 0.32)	-2.05 (1.91, -5.97, 1.22)	-0.3 (1.82, -3.71, 3.63)	-3.59 (3.01, -9.43, 0.622)	-4.12 (2.09, -8.17, -0.0397)	0.622 (0.551, -0.45, 1.73)	-0.537 (0.459, -1.55, 0.26)	0.681 (0.423, -0.0754, 1.59)	0.681 (0.576, -0.493, 1.72)	-0.219 (1.06, -2.31, 1.81)	-1.08 (0.799, -2.9, 0.194)	1.34 (0.845, -0.273, 2.98)	0.248 (0.369, -0.459, 1.01)	-15.3 (2.22, -18.8, -9.26)
	Apodemus (mouse)	Small	1.04	0.36 (0.121, 0.129, 0.602)	-0.286 (0.151, -0.593, -0.00086)	0.0339 (0.178, -0.327, 0.368)	-0.0945 (0.213, -0.524, 0.312)	-0.0749 (0.151, -0.373, 0.219)	-0.0758 (0.127, -0.33, 0.171)	-0.0215 (0.0921, -0.228, 0.129)	-0.117 (0.104, -0.351, 0.05)	0.0864 (0.0635, -0.0424, 0.208)	-1.61 (0.991, -3.88, -0.18)	-0.137 (0.114, -0.399, 0.0425)	0.0138 (0.0681, -0.132, 0.136)	-0.281 (0.146, -0.606, -0.0421)	0.0972 (0.0685, -0.0462, 0.223)	-0.572 (0.605, -1.79, 0.581)	-0.85 (0.349, -1.54, -0.172)	1.21 (0.377, 0.483, 1.96)	0.136 (0.189, -0.237, 0.507)	-0.323 (0.335, -0.992, 0.324)	0.534 (0.116, 0.307, 0.762)	0.271 (0.0875, 0.0983, 0.442)	-0.0235 (0.09, -0.202, 0.151)	-0.0208 (0.12, -0.256, 0.214)	-0.148 (0.233, -0.61, 0.306)	0.213 (0.135, -0.051, 0.481)	0.353 (0.198, -0.0245, 0.751)	0.125 (0.0896, -0.0508, 0.301)	-3.74 (0.108, -3.93, -3.5)
	Rattus (rat)	Medium	0.705	0.543 (0.42, -0.0603, 1.58)	-0.733 (0.485, -1.78, 0.0491)	-0.866 (0.741, -2.7, 0.211)	0.73 (0.637, -0.364, 2.13)	-0.288 (0.383, -1.15, 0.41)	-0.0283 (0.367, -0.88, 0.657)	-5.98 (1.79, -7.89, -1.3)	-2.53 (1.04, -5.77, -1.05)	0.373 (0.162, 0.014, 0.645)	-1.66 (0.664, -3.23, -0.397)	-1.87 (0.82, -3.27, -0.333)	-1.04 (1.02, -3.24, 0.0951)	-0.432 (0.372, -0.93, 0.139)	-0.0738 (0.249, -0.753, 0.259)	1.66 (2.27, -3.35, 5.83)	-7.11 (3.24, -13.3, -1.13)	5.62 (2.73, 0.819, 11.3)	-0.828 (1.58, -4.23, 2)	0.338 (1.28, -2.37, 2.82)	1.29 (0.668, 0.172, 2.83)	1.92 (0.714, 0.601, 3.5)	1.26 (0.517, 0.335, 2.41)	1.38 (0.697, 0.137, 2.78)	-0.827 (0.872, -2.63, 0.775)	-1.31 (0.904, -3.1, 0.179)	-1.7 (0.729, -3.13, -0.37)	0.197 (0.337, -0.457, 0.961)	-12 (4.33, -18.6, -6.43)
	Glirulus (dormouse)	Small	3.21	-0.186 (0.833, -1.24, 1.59)	-0.0252 (1.05, -2.32, 1.46)	0.824 (1.01, -1.04, 2.51)	-2.24 (1.89, -5.26, 0.853)	-4.37 (4.27, -12.8, -0.291)	0.931 (0.478, -0.0239, 1.77)	-8.47 (2.19, -12.6, -4.9)	-15.6 (1.66, -19.1, -12.6)	-1.66 (0.178, -1.79, -1.5)	-5.72 (1.42, -8.68, -3.18)	-4.97 (1.38, -6.32, -3.83)	-2.51 (1.02, -4.53, -0.707)	0.511 (0.0312, 0.456, 0.575)	-6.72 (1.46, -9.25, -4.93)	-3.92 (1.63, -7.21, -0.862)	-1.05 (0.643, -2.33, 0.196)	0.518 (0.584, -0.583, 1.76)	0.137 (0.32, -0.495, 0.76)	-1.33 (0.816, -2.97, 0.216)	0.613 (0.214, 0.193, 1.03)	-0.417 (0.795, -2.09, 0.736)	-0.56 (0.378, -1.36, 0.233)	-0.184 (0.206, -0.588, 0.22)	-0.0322 (0.735, -1.5, 1.37)	0.0791 (0.254, -0.422, 0.572)	1.42 (0.6, 0.255, 2.74)	-0.397 (0.412, -1.28, 0.341)	-12.4 (5.13, -23.9, -8.39)
Lagomorpha	*Lepus* (hare)	Medium	-1.84	0.148 (0.115, -0.0718, 0.381)	0.0988 (0.107, -0.11, 0.311)	-0.187 (0.135, -0.45, 0.0811)	-0.0877 (0.178, -0.438, 0.261)	0.101 (0.103, -0.107, 0.316)	0.237 (0.121, 0.00479, 0.481)	1.52 (0.666, 0.388, 3.02)	-0.0485 (0.0972, -0.242, 0.141)	0.0104 (0.0852, -0.143, 0.191)	-0.0464 (0.0501, -0.14, 0.0586)	-0.0313 (0.0504, -0.125, 0.0742)	0.0934 (0.0667, -0.0229, 0.239)	-0.0416 (0.0525, -0.144, 0.0629)	0.118 (0.0768, -0.0173, 0.283)	-4.39 (0.73, -5.88, -3.03)	-0.426 (0.279, -0.976, 0.122)	0.488 (0.299, -0.0987, 1.08)	-0.0506 (0.142, -0.329, 0.228)	-3.11 (0.389, -3.9, -2.38)	1.12 (0.111, 0.902, 1.34)	-0.231 (0.0768, -0.383, -0.0809)	0.531 (0.0696, 0.396, 0.668)	0.443 (0.112, 0.223, 0.661)	0.478 (0.14, 0.204, 0.755)	0.0637 (0.116, -0.163, 0.292)	0.577 (0.114, 0.356, 0.803)	0.129 (0.0603, 0.0116, 0.248)	1.69 (0.0984, 1.5, 1.88)
Carnivora	*Nyctereutes* (raccoon dog)	Medium	-0.82	-0.0918 (0.107, -0.298, 0.12)	0.207 (0.107, 0.000109, 0.42)	-0.281 (0.128, -0.531, -0.0293)	0.234 (0.178, -0.111, 0.588)	-0.105 (0.111, -0.325, 0.113)	0.211 (0.114, -0.00811, 0.44)	1.22 (0.531, 0.264, 2.4)	-0.0707 (0.0885, -0.242, 0.103)	0.00763 (0.0766, -0.132, 0.166)	0.329 (0.255, -0.00306, 0.929)	1.41 (0.514, 0.451, 2.49)	0.0286 (0.0675, -0.0962, 0.169)	-0.0398 (0.0528, -0.144, 0.0645)	0.000649 (0.0598, -0.109, 0.125)	-3.37 (0.623, -4.64, -2.21)	-0.335 (0.257, -0.843, 0.168)	0.28 (0.278, -0.265, 0.827)	-0.319 (0.128, -0.571, -0.0698)	-2.55 (0.336, -3.23, -1.91)	1.04 (0.101, 0.849, 1.25)	0.0652 (0.0719, -0.0753, 0.207)	0.416 (0.0649, 0.29, 0.545)	0.458 (0.103, 0.255, 0.661)	0.522 (0.137, 0.256, 0.792)	-0.115 (0.106, -0.324, 0.0934)	0.438 (0.108, 0.227, 0.65)	0.0377 (0.0581, -0.0759, 0.152)	1.24 (0.0832, 1.08, 1.4)
	Vulpes (fox)	Medium	-5.05	0.0939 (0.0902, -0.083, 0.271)	0.039 (0.0974, -0.151, 0.231)	-0.206 (0.11, -0.423, 0.0096)	0.159 (0.139, -0.112, 0.433)	-0.158 (0.1, -0.357, 0.0379)	0.0289 (0.0978, -0.163, 0.221)	0.277 (0.16, 0.0218, 0.647)	-0.0436 (0.068, -0.179, 0.0883)	-0.0415 (0.0581, -0.152, 0.0757)	-6e-04 (0.0556, -0.0979, 0.121)	0.00823 (0.045, -0.0757, 0.102)	-0.0556 (0.0449, -0.146, 0.0312)	0.0445 (0.0511, -0.0511, 0.15)	0.054 (0.0578, -0.0541, 0.173)	-4.23 (0.737, -5.71, -2.82)	-1.02 (0.256, -1.52, -0.518)	1.03 (0.279, 0.489, 1.59)	-0.114 (0.129, -0.367, 0.139)	-2.77 (0.378, -3.53, -2.05)	0.828 (0.0952, 0.643, 1.02)	-0.0264 (0.0758, -0.175, 0.122)	0.551 (0.0701, 0.416, 0.69)	0.382 (0.109, 0.167, 0.596)	0.331 (0.145, 0.047, 0.618)	-0.0976 (0.108, -0.309, 0.114)	0.459 (0.126, 0.213, 0.709)	0.125 (0.059, 0.00941, 0.241)	0.718 (0.0477, 0.623, 0.81)
	Martes (marten)	Medium	-4.41	-0.132 (0.112, -0.36, 0.0757)	0.0591 (0.0935, -0.124, 0.243)	0.0256 (0.13, -0.231, 0.281)	-0.115 (0.148, -0.41, 0.173)	0.164 (0.0955, -0.0206, 0.354)	-0.00237 (0.114, -0.23, 0.216)	0.177 (0.0926, 0.0194, 0.38)	-0.0323 (0.0552, -0.143, 0.0736)	-0.0627 (0.0547, -0.176, 0.0387)	-0.323 (0.149, -0.654, -0.0802)	4.76e-05 (0.0423, -0.085, 0.0818)	-0.0532 (0.0576, -0.176, 0.0498)	-0.00545 (0.0527, -0.115, 0.0925)	0.013 (0.0436, -0.0748, 0.0974)	-4.16 (0.683, -5.54, -2.86)	-0.116 (0.242, -0.593, 0.358)	0.442 (0.26, -0.0668, 0.956)	-0.127 (0.129, -0.38, 0.126)	-2.4 (0.351, -3.11, -1.73)	0.824 (0.088, 0.653, 0.998)	-0.188 (0.086, -0.359, -0.0217)	0.0287 (0.0642, -0.0973, 0.155)	0.0972 (0.0999, -0.0993, 0.293)	0.298 (0.156, -0.00805, 0.603)	0.104 (0.101, -0.095, 0.303)	0.488 (0.125, 0.245, 0.735)	-0.0234 (0.0573, -0.136, 0.0892)	-0.786 (0.0583, -0.904, -0.675)
	Mustela (mustelid)	Medium	-0.926	0.117 (0.0966, -0.0697, 0.31)	-0.0456 (0.0998, -0.241, 0.151)	-0.334 (0.118, -0.568, -0.103)	0.317 (0.156, 0.0137, 0.627)	0.0833 (0.107, -0.125, 0.295)	-0.0951 (0.0975, -0.286, 0.0972)	0.212 (0.111, 0.0247, 0.455)	-0.0109 (0.0624, -0.124, 0.121)	-0.0151 (0.0508, -0.112, 0.0886)	-0.156 (0.0502, -0.261, -0.0637)	0.898 (0.334, 0.275, 1.6)	0.0214 (0.0682, -0.0983, 0.168)	-0.0419 (0.0505, -0.139, 0.06)	-0.0565 (0.0526, -0.156, 0.0515)	-1.45 (0.428, -2.3, -0.622)	0.21 (0.263, -0.303, 0.728)	0.105 (0.28, -0.444, 0.655)	-0.278 (0.134, -0.544, -0.0164)	-1.27 (0.255, -1.78, -0.779)	0.629 (0.093, 0.448, 0.813)	0.0673 (0.0744, -0.0781, 0.214)	0.753 (0.0716, 0.615, 0.896)	0.544 (0.0971, 0.356, 0.737)	0.203 (0.144, -0.0781, 0.486)	0.259 (0.107, 0.0493, 0.471)	0.399 (0.109, 0.188, 0.614)	0.0943 (0.0587, -0.0205, 0.21)	0.551 (0.0518, 0.45, 0.653)
	Meles (badger)	Medium	-5.24	-0.173 (0.109, -0.399, 0.0351)	0.281 (0.108, 0.0705, 0.494)	-0.205 (0.142, -0.49, 0.068)	0.152 (0.155, -0.151, 0.458)	-0.174 (0.107, -0.389, 0.0337)	0.188 (0.114, -0.0387, 0.41)	0.0498 (0.0496, -0.0436, 0.151)	-0.0463 (0.0592, -0.18, 0.056)	-0.115 (0.0773, -0.291, 0.017)	-0.127 (0.093, -0.341, 0.0193)	-0.126 (0.0828, -0.312, 0.00922)	0.0064 (0.05, -0.0962, 0.106)	0.0811 (0.0491, -0.0176, 0.176)	-0.0519 (0.0518, -0.162, 0.0457)	-4.86 (0.907, -6.7, -3.14)	0.203 (0.283, -0.352, 0.76)	0.258 (0.316, -0.361, 0.88)	-0.0809 (0.152, -0.382, 0.218)	-3.08 (0.477, -4.05, -2.18)	0.668 (0.0998, 0.474, 0.866)	-0.142 (0.109, -0.36, 0.0665)	-0.0598 (0.0833, -0.224, 0.103)	0.398 (0.117, 0.17, 0.628)	0.0547 (0.234, -0.406, 0.511)	0.204 (0.123, -0.0369, 0.448)	0.29 (0.161, -0.0224, 0.608)	0.171 (0.0728, 0.0294, 0.315)	-1.05 (0.736, -2.5, 0.391)
	Ursus (bear)	Large	-4.31	-0.255 (0.195, -0.673, 0.0937)	0.146 (0.209, -0.276, 0.543)	0.367 (0.233, -0.0866, 0.824)	-0.852 (0.342, -1.55, -0.216)	0.196 (0.191, -0.179, 0.569)	0.0715 (0.201, -0.331, 0.458)	0.0891 (0.0534, -0.0155, 0.196)	0.0203 (0.0572, -0.0944, 0.139)	-0.162 (0.125, -0.44, 0.0483)	0.183 (0.0592, 0.0734, 0.308)	0.0861 (0.0916, -0.0948, 0.25)	-0.173 (0.16, -0.364, -0.0223)	-0.0983 (0.164, -0.461, 0.176)	-0.146 (0.161, -0.492, 0.126)	-5.27 (1.18, -7.66, -3.02)	-0.341 (0.396, -1.12, 0.433)	0.636 (0.444, -0.228, 1.51)	0.23 (0.185, -0.13, 0.596)	-2.93 (0.594, -4.13, -1.8)	1.22 (0.14, 0.948, 1.5)	-0.168 (0.168, -0.508, 0.149)	-0.327 (0.0982, -0.522, -0.136)	0.0672 (0.148, -0.223, 0.357)	0.305 (0.236, -0.157, 0.769)	0.0356 (0.164, -0.285, 0.358)	0.854 (0.254, 0.364, 1.36)	-0.0653 (0.093, -0.248, 0.117)	-3.85 (0.261, -4.32, -3.28)
Artiodactyla	*Sus* (wild boar)	Large	-1.99	-0.013 (0.133, -0.282, 0.241)	-0.0256 (0.126, -0.275, 0.223)	0.00989 (0.169, -0.328, 0.338)	-0.886 (0.255, -1.4, -0.396)	0.643 (0.164, 0.354, 0.993)	0.072 (0.146, -0.217, 0.359)	2.1 (0.552, 1.11, 3.28)	-0.167 (0.155, -0.456, 0.123)	-0.358 (0.13, -0.638, -0.134)	0.393 (0.192, 0.0612, 0.808)	-0.0391 (0.0506, -0.146, 0.0603)	-0.201 (0.0909, -0.404, -0.0498)	0.167 (0.0612, 0.0543, 0.297)	-0.000337 (0.0555, -0.107, 0.113)	-8.17 (1.44, -11.1, -5.48)	-0.208 (0.489, -1.17, 0.753)	0.423 (0.53, -0.616, 1.47)	-2.85 (0.448, -3.77, -2.01)	-5.13 (0.768, -6.72, -3.7)	1.67 (0.182, 1.34, 2.06)	-0.121 (0.12, -0.358, 0.113)	-0.14 (0.118, -0.373, 0.091)	0.904 (0.181, 0.557, 1.27)	0.948 (0.298, 0.377, 1.55)	-0.145 (0.199, -0.535, 0.248)	1.08 (0.217, 0.665, 1.52)	0.116 (0.0971, -0.0716, 0.31)	-6.22 (0.667, -7.26, -4.67)
	Cervus (sika deer)	Large	-2.69	-0.164 (0.227, -0.652, 0.245)	-0.178 (0.163, -0.505, 0.14)	-0.0989 (0.235, -0.59, 0.339)	-0.0997 (0.272, -0.642, 0.431)	0.215 (0.166, -0.105, 0.549)	-0.153 (0.209, -0.572, 0.249)	0.0929 (0.0592, -0.0234, 0.213)	-0.181 (0.128, -0.484, 0.00866)	-0.032 (0.108, -0.272, 0.151)	-0.0838 (0.0693, -0.238, 0.0369)	-0.0669 (0.0986, -0.285, 0.0976)	-0.197 (0.137, -0.519, 0.0139)	0.0486 (0.069, -0.0922, 0.182)	-0.00826 (0.0633, -0.137, 0.115)	-1.31 (0.723, -2.76, 0.0841)	-1.31 (0.498, -2.31, -0.35)	2.53 (0.545, 1.5, 3.65)	-0.984 (0.268, -1.52, -0.468)	-1.57 (0.446, -2.48, -0.724)	1.34 (0.165, 1.04, 1.69)	-0.239 (0.134, -0.507, 0.0194)	-0.0163 (0.102, -0.217, 0.186)	0.481 (0.147, 0.2, 0.778)	0.413 (0.237, -0.0489, 0.883)	0.193 (0.183, -0.163, 0.556)	0.996 (0.218, 0.582, 1.44)	0.162 (0.0901, -0.0126, 0.342)	-2.49 (0.13, -2.75, -2.25)
	Capricornis (serow)	Large	-4.7	-0.309 (0.212, -0.772, 0.07)	-0.396 (0.241, -0.897, 0.0572)	0.247 (0.204, -0.164, 0.68)	-0.704 (0.355, -1.44, -0.044)	0.562 (0.249, 0.0806, 1.06)	0.354 (0.195, -0.0298, 0.738)	0.00193 (0.135, -0.31, 0.213)	-0.00871 (0.0929, -0.208, 0.156)	-0.247 (0.177, -0.658, 0.0359)	-0.216 (0.326, -1.04, 0.161)	-0.0323 (0.0796, -0.204, 0.118)	-0.067 (0.0777, -0.241, 0.0759)	0.0606 (0.0795, -0.105, 0.214)	-0.154 (0.184, -0.565, 0.142)	-8.38 (1.79, -12.2, -5.18)	-0.402 (0.5, -1.39, 0.578)	2.11 (0.588, 1, 3.32)	0.317 (0.262, -0.184, 0.865)	-5.08 (0.979, -7.21, -3.35)	1.61 (0.219, 1.21, 2.08)	0.0445 (0.178, -0.312, 0.386)	-0.528 (0.146, -0.831, -0.252)	-0.0407 (0.208, -0.454, 0.364)	1.1 (0.423, 0.284, 1.95)	0.465 (0.213, 0.059, 0.899)	1.48 (0.375, 0.779, 2.25)	-0.083 (0.13, -0.338, 0.172)	-1.83 (1.17, -4.17, 0.454)

*1 Ohdachi et al. 2009. Small: less than 100 g, Medium: between 100 g and 10 kg, Large: over 10 kg. Size classes were defined according to Prothero (2015).
